# Phenylketonuria (PKU) Urinary Metabolomic Phenotype Is Defined by Genotype and Metabolite Imbalance: Results in 51 Early Treated Patients Using Ex Vivo ^1^H-NMR Analysis

**DOI:** 10.3390/molecules28134916

**Published:** 2023-06-22

**Authors:** Claire Cannet, Allan Bayat, Georg Frauendienst-Egger, Peter Freisinger, Manfred Spraul, Nastassja Himmelreich, Musa Kockaya, Kirsten Ahring, Markus Godejohann, Anita MacDonald, Friedrich Trefz

**Affiliations:** 1 Bruker Biospin, 76275 Ettlingen, Germany; claire.cannet@bruker.com (C.C.);; 2 Kennedy Centre, Center for PKU, 2600 Glostrup, Denmark; 3 Department of Pediatrics, School of Medicine, University of Tübingen, 72074 Tübingen, Germany; 4 CEGAT, Human Genetic Institute, 72076 Tübingen, Germany; 5 Private Pediatric Practice, 68307 Mannheim, Germany; 6 Dietetic Department, Birmingham Children’s Hospital, Birmingham B4 6NH, UK; 7 Metabolic Consulting Reutlingen, 72766 Reutlingen, Germany

**Keywords:** phenylketonuria, metabolomics, Ex Vivo 1H-NMR analysis spectroscopy, genotype, pathogenesis

## Abstract

Phenylketonuria (PKU) is a rare metabolic disorder caused by mutations in the phenylalanine hydroxylase gene. Depending on the severity of the genetic mutation, medical treatment, and patient dietary management, elevated phenylalanine (Phe) may occur in blood and brain tissues. Research has recently shown that high Phe not only impacts the central nervous system, but also other organ systems (e.g., heart and microbiome). This study used ex vivo proton nuclear magnetic resonance (^1^H-NMR) analysis of urine samples from PKU patients (mean 14.9 ± 9.2 years, *n* = 51) to identify the impact of elevated blood Phe and PKU treatment on metabolic profiles. Our results found that 24 out of 98 urinary metabolites showed a significant difference (*p* < 0.05) for PKU patients compared to age-matched healthy controls (*n* = 51) based on an analysis of urinary metabolome. These altered urinary metabolites were related to Phe metabolism, dysbiosis, creatine synthesis or intake, the tricarboxylic acid (TCA) cycle, end products of nicotinamide-adenine dinucleotide degradation, and metabolites associated with a low Phe diet. There was an excellent correlation between the metabolome and genotype of PKU patients and healthy controls of 96.7% in a confusion matrix model. Metabolomic investigations may contribute to a better understanding of PKU pathophysiology.

## 1. Introduction

Phenylketonuria (PKU; OMIM#261600) is a rare metabolic disorder caused by mutations in the phenylalanine hydroxylase (PAH) gene. Depending on the severity of the genetic mutation, patient adherence to dietary phenylalanine (Phe) restriction, and the efficacy of medical treatments, elevated blood Phe may occur in blood and brain tissues. If not diagnosed and treated early in the neonatal screening program, this elevated blood Phe can cause intellectual disability, behavioral and psychiatric problems, microcephaly, motor deficits, eczematous rash, autism, seizures, and developmental problems. The monitoring of treatment in PKU patients is based on blood Phe using target Phe levels for different age groups [1,2]. Up to now, the traditional explanation for the toxic effect of elevated Phe on brain development and brain function has been neurotransmitter depletion [3,4] and amino acid imbalances [5]. However, in addition to these neuropathological effects, elevated blood Phe has been observed to adversely affect other organ systems (e.g., eye [6,7] and heart [8,9,10]). A recent review has proposed that the explanation for peripheral organ involvement in the pathology of PKU involves energy dysregulation, oxidative stress [11], and the gut microbiome [12]. Dietary Phe restriction and/or the special semisynthetic, low-Phe medical food diet intended to benefit patients with PKU may also impact normal metabolic processes. Therefore, our understanding of the pathology of PKU is expanding to include other, more complex, dysregulated pathways.

Different analytical techniques have been used to investigate the adverse impact of elevated blood Phe on metabolic pathways, substances, and organ systems [10,12,13,14,15,16]. Analyzing the quantity of small molecules (i.e., the metabolome) may lead to a better insight into the impact of elevated Phe on pathways and organ systems. In addition, methods using untargeted and targeted metabolomics [17,18,19,20] may improve the diagnosis and treatment of inborn errors of metabolism. Progress in nuclear magnetic resonance spectroscopy (NMR) technology in analyzing the metabolome may offer an additional method to study metabolic diseases [21,22,23,24,25].

The aim of this study is twofold: (1) to investigate if the urinary metabolome correlates with the severity of the genetic defect (genotype) and (2) to reveal the impacts of elevated blood Phe and/or dietary and medical treatments on metabolic pathways and the gastrointestinal microbiome using targeted and untargeted metabolomic analysis in the urine.

## 2. Results

### 2.1. Patient Characteristics

All patients were diagnosed and treated shortly after birth. The age range was 0.25–33 years for PKU patients and age-matched healthy controls. Table 1 shows that there was no significant difference for age, gender, or for urinary creatinine in PKU patients and age-matched controls.

### 2.2. Metabolome and Genotype

**Figure 1:**
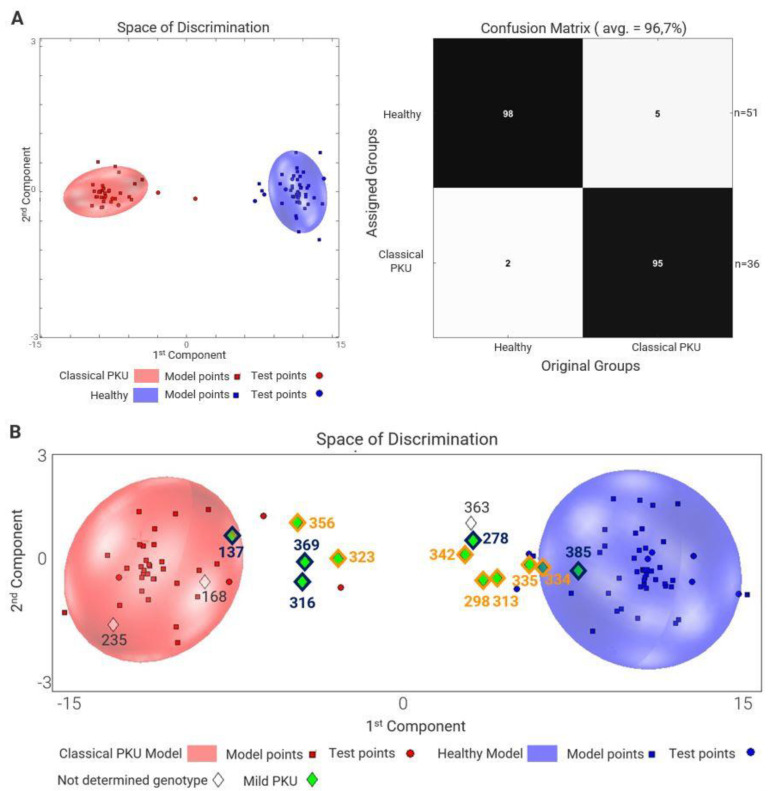
(**A**) Discrimination between classical PKU (cPKU) and healthy controls. PCA/CA/MCCV classification shows clear discrimination between cPKU (red ellipsoid) and an age-matched healthy control group (blue ellipsoid) with confusion of 96.7%. The space of discrimination is one representation of the modelling samples in two dimensions. The ellipsoids represent the 95% percentile of the model. (**B**) Prediction of mild PKU (mPKU) and non-determined genotype patients into the cPKU and healthy control model. Patients (except ID 137) with diamonds had a genotype phenotype value (GPV) of >2.7–6.9 and a phenotype of mPKU. Patients with green/black diamonds are not treated with sapropterin; green/yellow diamonds are treated. Patients with open diamonds have no classification because genotype was not available. Patient 385 has a phenotype between mPKU and hyperphenylalaninemia (HPA) according to [26].

Figure 1 shows a clear discrimination between the spectroscopic urinary fingerprints of the classical PKU (cPKU) group (red ellipsoid, *n* = 36) and healthy controls (blue ellipsoid, *n* = 51). The mild PKU (mPKU) patients (*n* = 12) are predicted between the cPKU group and healthy controls. Because of the small numbers, no differences were calculated in those who were treated with sapropterin (sapropterin dihydrochloride/tetrahydrobiopterin/BH_4_), but they are more similar to the healthy control group. All the 36 cPKU patients had a genotype/phenotype value (GPV) of 0–2.7; PKU patients with GPV > 2.7–6.9 are outside and between cPKU and healthy controls.

There are two exceptions. For patient ID 137, the urinary fingerprint of this patient is predicted in the cPKU group, even though he is defined as mPKU. However, he was not treated with sapropterin, and his plasma Phe level was 1118 µmol/L. For patient ID 385, the NMR spectrum is predicted to be in the healthy control group. His phenotype was evaluated between mPKU and mild hyperphenylalaninemia (MHPA) [26]. He was not treated with sapropterin, but with a low Phe-restricted diet. Three patients represented by a diamond (◊) symbol in Figure 1B had blood Phe levels and/or genetic data that were not meaningful for a clear phenotype definition: two of them are predicted in the cPKU group, and one between healthy controls and cPKU group. Thus, the number of cPKU was set to 38, and of mPKU to 13.

### 2.3. Serum Phenylalanine in PKU Patients

**Figure 2:**
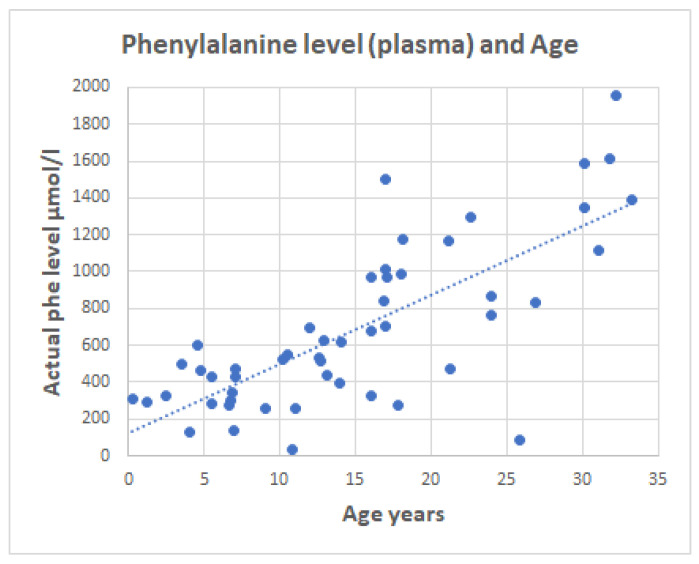
Serum phenylalanine (Phe) level (µmol/L) by age for patients with PKU (*n* = 51).

Week 1 and week 2 serum Phe concentrations were not significantly different, so only serum Phe data collected at week 1 was used in this study. As expected, serum Phe concentrations are lower for younger patients. Figure 2 shows a trend of increasing blood Phe with age; this is a phenomenon previously observed and attributed to gradual patient non-compliance to dietary Phe restriction [27].

**Figure 3:**
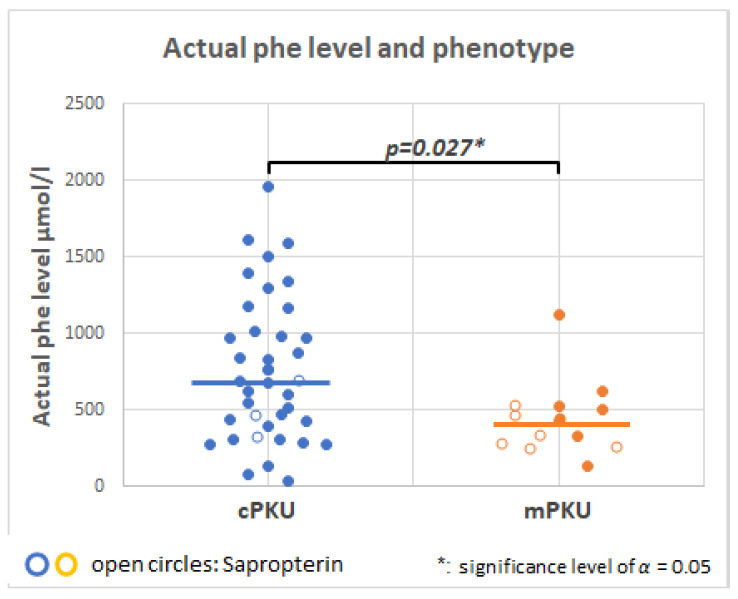
Serum phenylalanine (Phe) level (µmol/L) by classical PKU (cPKU, *n* = 38) or mild PKU (mPKU, *n* = 13) phenotype. Patients indicated with open circles are treated with sapropterin dihydrochloride (tetrahydrobiopterin [BH_4_]).

Figure 3 shows that there is a significant difference in serum Phe when cPKU (mean 777 + 467 µmol/L) and mPKU (mean 421 ± 255 µmol/L) phenotypes are compared. For both phenotypes, sapropterin (shown as open circles) may help lower blood Phe. There was one outlier with a BH_4_ responsive genotype, but who was not treated with sapropterin and had a blood Phe level of 1118 µmol/L (ID 137, Figure 1).

Table 2 summarizes PKU patient characteristics showing serum Phe levels, genotypes with GPV, total natural protein intake (g/kg bodyweight/day), and sapropterin treatment. PKU treatment is a Phe-restricted diet (*n* = 31), sapropterin supplementation (*n* = 9), and no dietary treatment except supplementation with large neutral amino acids (*n* = 6). Mean patient age is 14.9 years (range–0.25 to 33 years).

Table 2 also shows the mutation analysis for the *PAH* gene for both alleles (*n* = 45). In the six other patients, phenotype was derived from the metabolome (ID 168, 235, 362; Figure 1), serum Phe levels > 1200 µmol/L (ID 143 and 129), and in patient ID 385 from [26]. In patient ID 143, the terminology reported was unusual (c.−473−? 168+?du), but may be a deletion leading to a null mutation (Nenad Blau, personal communication).

### 2.4. Dietary Intake

**Figure 4:**
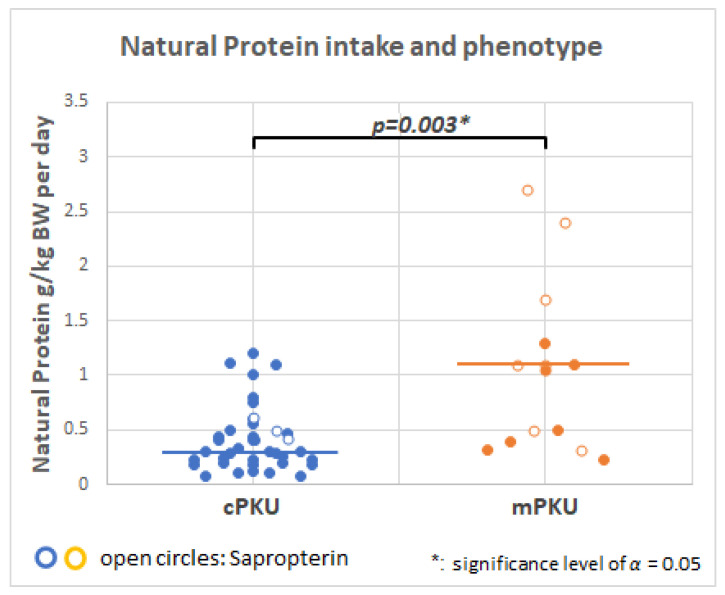
Patients with mild PKU (mPKU) have a significantly higher natural protein intake per day than patients with classical PKU (cPKU). A few patients were on sapropterin treatment (mPKU, *n* = 7 and cPKU, *n* = 3, open circles).

Figure 4 shows that average natural protein intake (g/kg bodyweight/day) for cPKU patients is significantly lower than for mPKU patients. Dietary protein intake was more variable for patients with mPKU than cPKU patients. A few patients with mPKU (*n* = 7) and cPKU (*n* = 3) are on sapropterin treatment; these *n* = 3 patients had higher GPV values (see Table 2) but are still defined as cPKU [28].

### 2.5. NMR Targeted Analyses

A total of *n* = 149 metabolites (Supplementary Table S1) were analyzed using high resolution ^1^H NMR, which are quantified automatically [29]. A subset (*n* = 98) of metabolites, for controls and patients, had sufficient data (>10) for statistical analysis (i.e., they were above the detection limit of the method). All others were excluded from subsequent analysis.

Table 3 shows the *n* = 24 urinary metabolites that were significantly different (*p* < 0.05) compared to age-matched healthy controls (*n* = 51). They are ranked in Table 3 according to their fold change. These metabolites represent different substance classes, pathways, and origin.

### 2.6. Metabolites Linked to Energy Metabolism

#### 2.6.1. *N*-Methyl-2-pyridone-5-carboxamide (2PY), HMDB0004193, Fold Change 1.728

**Figure 5:**
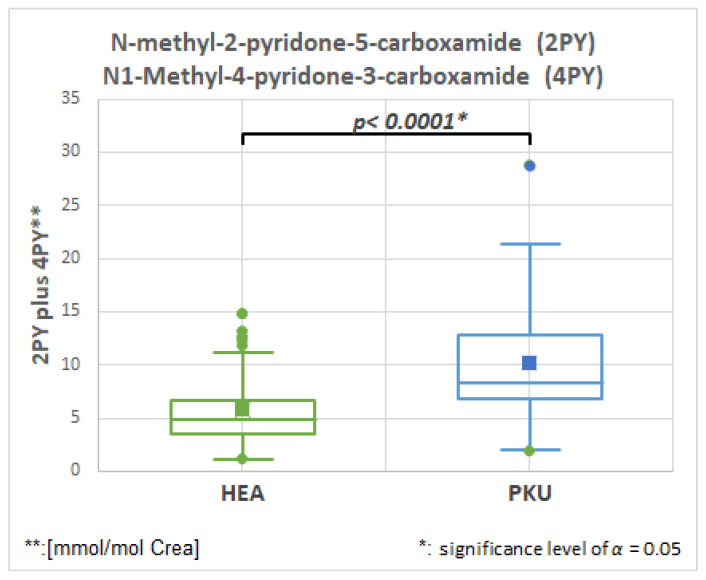
NADH degradation products, *N*-methyl-2-pyridone-5-carboxamide (2PY) and *N*1-Methyl-4-pyridone-3-carboxamide (4PY) are elevated in PKU patients in comparison to healthy controls (HEA).

Figure 5 and Table 3 show that the NADH degradation products, *N*-methyl-2-pyridone-5-carboxamide (2PY) and *N*1-Methyl-4-pyridone-3-carboxamide (4PY) are significantly elevated in PKU patients.

The initial automatically quantified substance analysis appeared to detect allopurinol, a common drug for hyperuricemia, in all urine samples, even though none had received the drug. Further analysis using ultraperformance liquid chromatography mass spectrometry (UPLC/MS) and confirmation by NMR using proton one-dimensional and proton-carbon heteronuclear two-dimensional spectroscopy (Supplementary Figure S1 and text) suggested that the detected compounds are two structurally similar pyridine metabolites: *N*-methyl-2-pyridone-5-carboxamide (2PY), HMDB0004193, and *N*1-Methyl-4-pyridone-3-carboxamide (4PY), which are both end products of nicotinamide-adenine dinucleotide (NAD) degradation and are observed in higher concentrations in uremic patients [30].

#### 2.6.2. 1-*N*-Methylnicotinamide, HMDB0000699, Fold Change 1.459

Table 3 and Supplementary Figure S2 show that 1-*N*-Methyl nicotinamide concentrations are higher in patients with PKU than healthy controls. 1-*N*-Methyl nicotinamide is found in various plants, but also in bodily fluids. More recent studies in rats revealed a possible link to irritable bowel syndrome and dysbiosis [31]. Other investigators have identified 1-*N*-Methyl nicotinamide as having an important role in NAD metabolism with regard to cellular energy and “healthy aging” [32]. It has also been found to be predictive of various diseases such as polycystic kidney disease [33].

#### 2.6.3. Oxaloacetic, HMDB0000223, Fold Change 1.731

**Figure 6:**
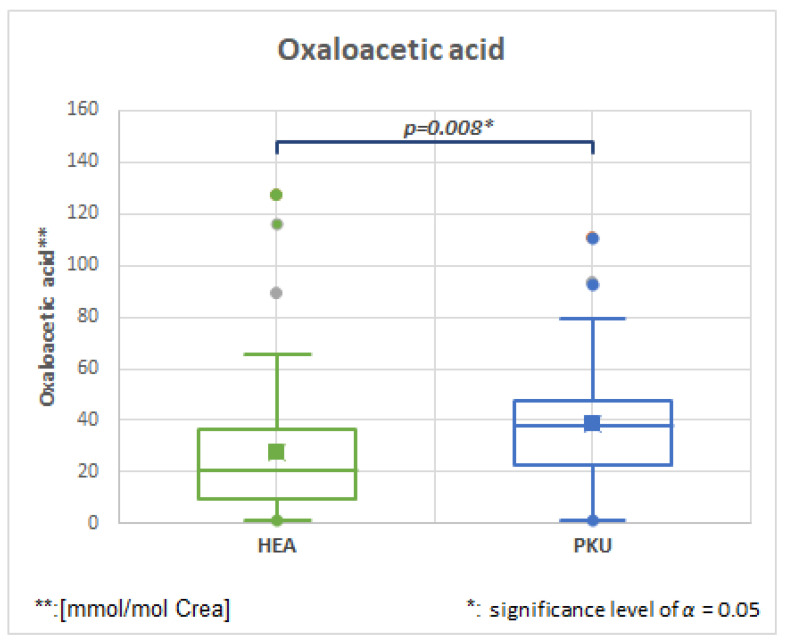
Oxaloacetic acid is significantly elevated in PKU patients in comparison to healthy controls (HEA).

Figure 6 and Table 3 show that oxaloacetic acid is significantly elevated in PKU patients. Oxaloacetic acid is an intermediate of the citric acid cycle (TCA), and its role in energy metabolism in the PKU mouse model has been shown recently [11,34]. Increased oxaloacetic concentrations may indicate an impairment of glucose-6-phosphate-dehydrogenase by high phenylpyruvate and reduced pyruvate in the TCA cycle.

#### 2.6.4. Creatine HMDB0000064 and Guanidinoacetic Acid HMDB0000128, Fold Change 0.796 and 0.796

**Figure 7:**
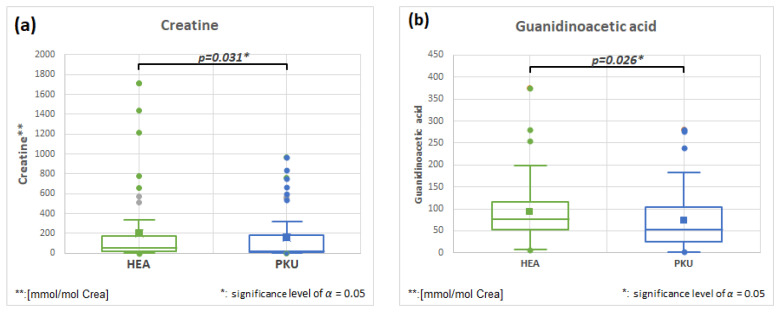
Creatine (**a**) and guanidinoacetate (**b**) are slightly decreased in PKU patients in comparison to healthy controls (HEA).

Creatine plays an important role in energy metabolism. One precursor of endogenous synthesis is guanidinoacetic acid. Figure 7a,b show that both metabolites are significantly different and slightly decreased in PKU patients. Several inborn errors of metabolism are due to creatine deficiency [35]. The sources of creatine are mainly foods rich in meat, but creatine is also synthesized in the liver via glycine and arginine [35]. It is also decreased in the serum of PKU patients [36].

### 2.7. Metabolites Related to Gut and Dietary Treatment

#### 2.7.1. Tartaric Acid HMDB0000956 and L-Citramalic HMDB0000426 Acid (Fold Change 2.665 and 1.721)

**Figure 8:**
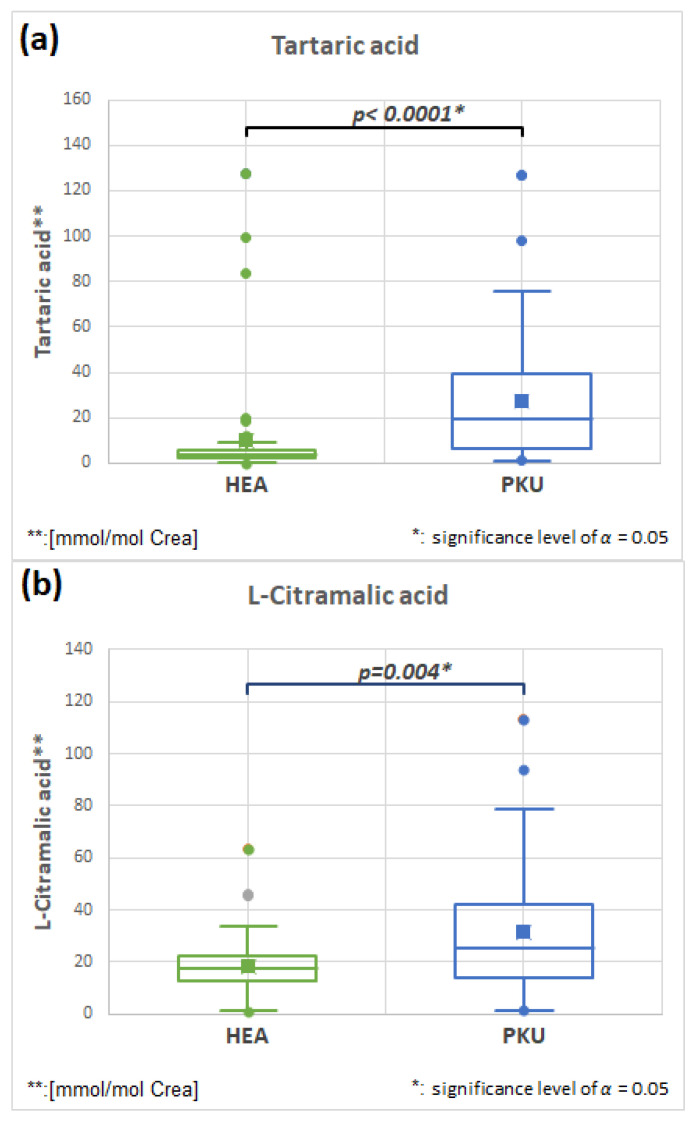
Tartaric (**a**) and L-Citramalic (**b**) acid are significantly elevated in PKU patients in comparison to healthy controls (HEA).

Figure 8a,b, and Table 3 show that tartaric and L-citramalic acid are both significantly elevated in PKU patients. Each of these substances are mainly produced by bacteria in the microbiome, and have been described as markers of dysbiosis [12,37].

#### 2.7.2. Acetic Acid, HMDB0000042, Fold Change 1.931

Acetic acid is significantly elevated in patients with PKU (Supplementary Figure S2). The role of acetic acid produced by bacteria in the gut has recently been investigated in animal models [38]. It may also play a role in irritable bowel syndrome [39]. Acetic acid is present in very low concentrations in the cell, but greatly increased by bacteria in urinary tract infections [40], indicating that acetic acid is mainly derived from bacteria. It is elevated in PKU patients (see Table 3 and Supplementary Figure S2).

#### 2.7.3. Allantoin, HMDB00462, Fold Change 0.440

**Figure 9:**
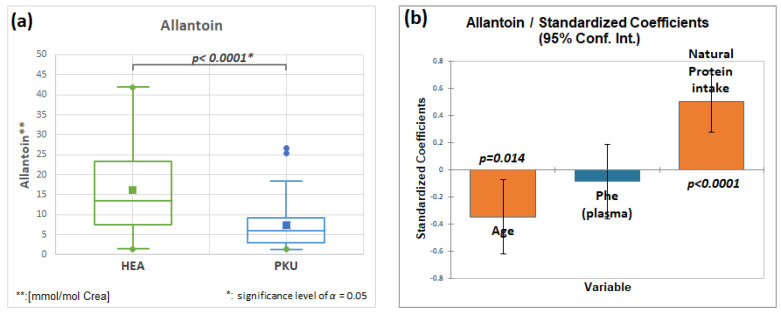
(**a**) Allantoin is decreased in PKU patients, (**b**) Multifactorial analysis of allantoin using standard coefficient of variation: negative with age (*p* < 0.0001), and phenylalanine (Phe) level in plasma (not significant), and positive with natural protein intake (*p* < 0.0001).

**Figure 10:**
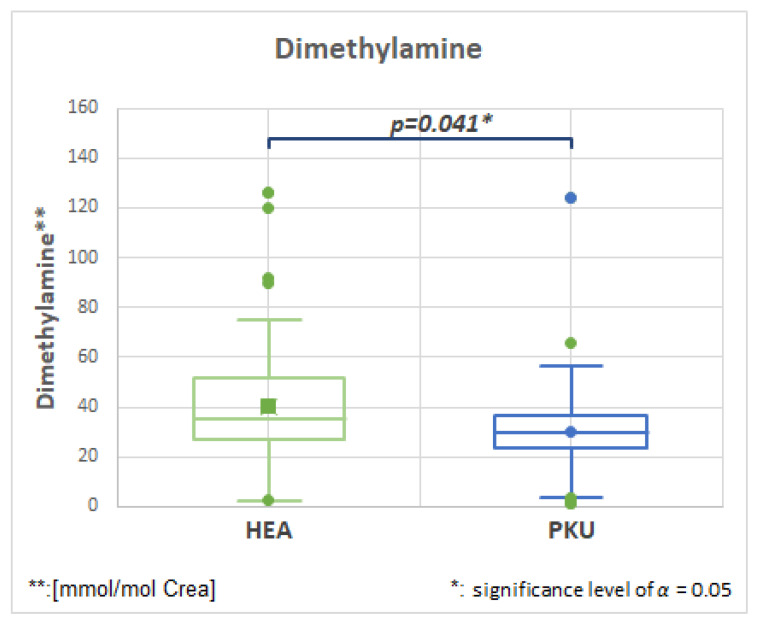
Dimethylamine is decreased in PKU patients in comparison to healthy controls (HEA).

Figure 9a and Table 3 show that allantoin is significantly decreased in patients with PKU. Allantoin is generated by reactive oxygen species from uric acid [41]. In patients with PKU, the cause of the decrease in Figure 9a may be due to their mainly vegetarian diet [42]. This is supported by a multifactorial regression analysis; natural protein intake has a significant positive effect (Figure 9b) on allantoin excretion. Figure 10 shows that a standardized coefficient analysis of metabolites demonstrated a negative correlation with subject age (−0.568, *p* < 0.0001).

#### 2.7.4. Dimethylamine, HMDB00087, Fold Change 0.753

Figure 10 and Table 3 show that dimethylamine is decreased in patients with PKU compared to healthy controls. Dimethylamine is converted from trimethylamine and is mainly found after ingestion of fish and seafood [43] and should not be consumed by PKU patients on a protein-restricted diet.

#### 2.7.5. 2-Furoylglycine, HMDB0000439, Fold Change 0.661

2-Furoylglycine is significantly elevated in patients with PKU (Supplementary Figure S2, Table 3), and may be derived from furan derivatives which are found in food prepared with strong heating. It is generally not found in urine from breastfed children but is found in that of formula-fed children [44]; it may be caused by a semisynthetic diet.

### 2.8. Other Metabolites Showing Significant Differences (Supplementary Figure S2)

#### 2.8.1. Amino Acids

Table 3 shows several amino acids (e.g., glycine, valine, methionine), in addition to those amino acids related to Phe metabolism, that are significantly increased in PKU patients. Interpretation of differences between groups is difficult and may be due to supplementation of the low protein diet with amino acid mixtures/tablets low or free of Phe. Elevated glycine is found in several inborn errors of metabolism; the highest levels are found in non-ketotic and ketotic hyperglycinemia due to an impaired function of the glycine cleavage enzyme (for overviews, e.g., www.metagene.de, metabolite “glycine”, accessed on 1 June 2023).

#### 2.8.2. Organic Acids

In addition to the metabolites derived from elevated blood Phe, Table 3 shows two important organic acids that are elevated in patients with PKU (i.e., 2-Hydroxyisovaleric acid and 3-Methylglutaconic acid). The organic acid 2-Hydroxyisovaleric acid (HMDB0001863) is found to be elevated in lactic acidosis and several organic acidemias, such as propionic acidemia, and multiple carboxylase deficiency. The organic acid 3-Methylglutaconic acid is linked to several inborn errors of energy metabolism and (in high concentrations) may be a “metatoxin” (HMDB0000522).

Acetoacetate is one of the ketone bodies, and is elevated in starvation and decompensated diabetes mellitus. There is only a slight elevation in urine (Table 3, Supplementary Figure S2) compared to normal controls. The incorporation in vivo of [14C]acetoacetate into cerebral lipids was decreased by Phe in a rat model [45] of HPA.

#### 2.8.3. Phenylalanine and Phenylalanine Derived Oxidation Products

Beside Phe, phenylpyruvic acid, D-mandelic acid, and phenylacetic acid could be detected with significant differences compared to the control samples (see Table 3 and Supplementary Figure S2). Interestingly, phenylpyruvate has the highest effect in fold change (6.853). Neopterin is elevated in patients with PKU and high blood Phe [46].

## 3. Discussion

We applied ^1^H NMR ex vivo analysis to a cross-sectional cohort study of PKU patients in whom genetic, biochemical, and dietary regimens were carefully documented. For the first time, we correlated the metabolome with genetic data and distinguished the effect of genotype, medical treatment, and normal controls. In addition, Figure 1 shows that the genetic profiles of mPKU patients (reflecting the metabolome with a GPV > 2.7–6.9) could be observed to reside between those of healthy controls and cPKU patients. With the exception of patient ID 137, we hypothesize that the uniqueness of the urinary metabolome for mPKU may be due to several possible factors: (1) an altered excretion of Phe and its degradation end products [19]; (2) the influence of a low-Phe diet [15]; (3) various other influencers (e.g., drugs) (Supplementary Figure S2); and (4) the secondary effects of elevated Phe on various pathways and the gastrointestinal microbiome [13].

Urine is one of the most complex biological fluids. Therefore, we added a targeted urinary analysis comprising quantification of 98 metabolites using ex vivo ^1^H NMR, a method with both advantages and disadvantages [26]. In another similar study, it was not possible to draw conclusions for the treatment quality of a (small) set of PKU patients by analyzing blood with a UPLC/MS/MS method [47]. In contrast, the present study was able to successfully identify PKU patients using the urinary metabolome. We demonstrated an excellent correlation between the metabolome and genotype of PKU patients and healthy controls (96.7%) in a confusion matrix model. One may speculate that application of such a model would support treatment monitoring in PKU patients.

Multiple metabolic effects of different pathways in treated PKU patients have been shown in plasma and urine by other investigators [13]. The advantage of our study that it uses a method that enables quantification of a wide range of different substance classes, indicating involvement of two new aspects in the pathophysiology of PKU: energy metabolism and dysbiosis.

Evidence for dysbiosis in patients with PKU can be found in the significant alterations in the 1-*N*-methylnicotinamide, tartaric acid, and L-citramalic acid concentrations. The pathophysiology of dysbiosis has been confirmed by investigators of other metabolic [48] and neurological diseases (e.g., Alzheimer’s disease [49], Parkinson’s disease [50]). The microbiome and small molecules also have roles in the aging process [51]. For PKU, the microbiome and dysbiosis may contribute to a more complete understanding of PKU pathophysiology [52]. Of course, our metabolic study is restricted only to a possible dysbiosis reflected by altered concentrations of metabolites derived from bacterial origin. Dietary management in PKU patients may modulate the composition of gut bacteria and contribute to its metabolomic profile. There is less bacterial diversity in PKU compared to healthy controls [53]. In addition, a decrease in fecal butyrate content in PKU patients has been observed [54].

A second finding of our study is that dysregulation of energy metabolism may be an important new aspect of PKU pathophysiology. We revealed an alteration in a metabolite associated with the TCA (oxaloacetic acid), and thus possible mitochondrial dysfunction. Energy dysfunction in PKU has been observed in animal studies [11]. Energy dysregulation in PKU could also explain the impact of elevated Phe on brain tissues, as well as other affected organs such as the heart [10,55], eyes [56,57], and on renal dysfunction [58,59,60].

### 3.1. Phenylalanine and Phenylalanine Metabolites

The main metabolic pathway of Phe is protein synthesis and the production of tyrosine in the liver. In the case of elevated Phe, the transamination pathway to phenylpyruvate, phenyllactate, and phenylacetate can be demonstrated using measurement of these and other substances in urine. Previously, there has been scientific debate about the potential toxicity of one of these metabolites and resultant brain damage in untreated PKU (see extensive discussion in [61]).

### 3.2. Role of Natural Low Protein Intake on the Metabolome

It has been suggested that natural protein is superior to synthetic protein, and that dietary Phe intake should increase stepwise whenever blood Phe is in the target range [62]. However, it is acknowledged that medical management of PKU is complex and multifactorial [2,63]. Although the number of patients is small, our study observed the expected higher intake of natural protein in mPKU compared to cPKU patients (Figure 4), which may increase with BH_4_ medication in some patients. One study showed that a natural protein intake of >0.5 g/kg/day was associated with improved body composition [64].

### 3.3. Limitations

**Figure 11:**
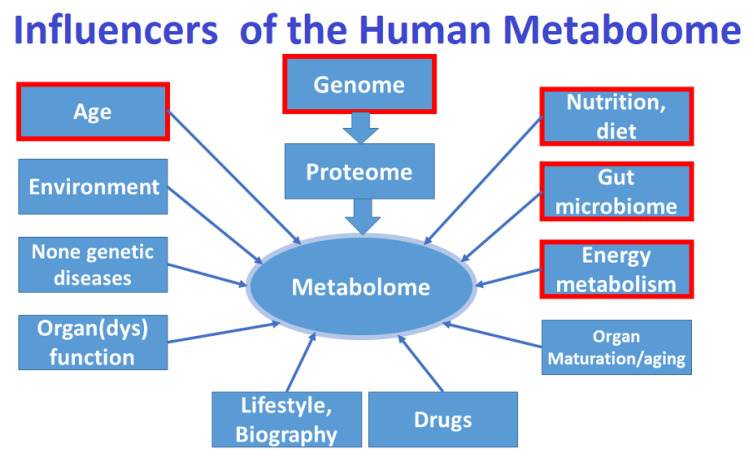
Possible influences on the human metabolome. In red are factors related to phenylketonuria and investigated in our study.

This study has several limitations. First, only 24 of 98 metabolites were found to be different in a small cohort of patients with PKU compared to an age-matched control group. Other metabolically important yet unidentified compounds may make up an important part of the PKU metabolome. Second, although the ex vivo ^1^H NMR method used in this study is highly quantitative and reproducible, this method has a higher detection limit than UPLC- or GC-MS/MS. The number of metabolites used for the automatic analysis is limited to *n* = 149, so other detectible substances may have been unobserved using full automation (according to standard procedures with the Bruker IVDr System). There may be other possible metabolites that contribute to the differences between the PKU and control group, as also shown by the metabolomic analysis, resulting in a good separation between patients and controls (Figure 1a,b). In addition, the analysis of urinary metabolites may not be representative of other important metabolites found in other body fluids (e.g., plasma or cerebral spinal fluid) [3,4,14]. Finally, Figure 11 shows that there are many potential influences on the metabolome. An important potential influencer is age, which is demonstrated in Supplementary Figure S3 (for patients and controls) and in Figure 5 (for patients using multifactorial analysis). Further influences in patients with PKU apart from age may be serum Phe and natural protein intake (Supplementary Figure S4). Even the use of multifactorial analysis (Supplementary Figure S5) makes it difficult to differentiate between genetic defects and natural effects (e.g., aging). Whether or not a patient with PKU is more at risk of non-healthy aging [65] should be investigated in future studies.

## 4. Materials and Methods

### 4.1. Study Participants

This cross-sectional study with PKU patients (*n* = 51) and age-matched healthy controls (*n* = 51) was approved by institutional ethics committees (Table 1). Patients with PKU were recruited from various private practice clinics and provided written informed consent. Guardians provided written consent for minors to participate. Healthy controls were recruited from otherwise routine investigations of healthy children/adults in private practice after written informed consent under ethical approval (EK LAEK BW, F-2013-006).

### 4.2. Sample Collection

Spontaneous morning urine samples (3–10 mL) collected from patients with PKU and healthy controls, and 1 mL aliquots were stored frozen at −20 °C prior to measurement. In patients, blood for Phe measurements was drawn in the morning at two weekly intervals. Serum was frozen at −20 °C prior to measurement, as previously described [26].

### 4.3. Blood Phenylalanine Analysis

Blood Phe in serum was measured according to local routine methods [66]. Mutational analyses [26] and classification of patients with PKU or HPA were performed as previously described [28,29]. Stratification of patients according to their genotype was performed using the genotype/phenotype value (GPV) [28].

### 4.4. NMR Analysis

Urine samples were first prepared according to standard procedures as previously described [67]. Frozen urine samples were thawed at 4 °C and shaken before use. A volume of 0.9 mL of urine was added into another cryovial of 0.1 mL potassium phosphate buffer (pH 7.4) containing trimethylsilylpropionic acid-d sodium salt (TSP) and sodium azide. The mixture was homogenized, and 0.6 mL was transferred to a 5 mm NMR tube for analysis and placed in a cooled sample changer. Samples were then measured, in full automation and according to standard procedures, using a Bruker IVDr System, as previously described [29,68].

### 4.5. Targeted NMR Analysis

The absolute and relative concentrations of 149 metabolites (Supplementary Table S1) were calculated automatically from all urinary NMR spectra using the B.I.QuantUR analysis tool. Only 98 of 149 metabolites were used for analysis because the concentrations of the other metabolites were below the limit of detection. Concentrations are given in mmol/mol creatinine as the urine collection was done on spot urine.

### 4.6. Untargeted NMR Analysis

Patients were stratified according to their GPV: (1) classical PKU (cPKU) patients (GPV 0–2.7) and (2) mild PKU/HPA (mPKU) patients (GPV 2.8–7). There was only one patient (patient ID 385) whose Phe value was borderline hyperphenylalaninemia (HPA) and mPKU. Individual GPV values were determined using the BioPKU database (www.biopku.org, accessed on 1 June 2023). Where GPV was not available because of missing mutational information, blood Phe concentrations of >1200 µmol/L were defined as classical PKU.

For the untargeted approach, we used the PCA/CA/k-NN MCCV analysis. We were able to create a classification model of cPKU (*n* = 36) vs. healthy controls (*n* = 51), and projected the undetermined genotype (*n* = 3) and the mPKU (*n* = 12) in the model. The steps performed have already been described by Assfalg et al. [69] and Bernini et al. [70].

Spectral binning: Prior to further postprocessing, spectral intensity was scaled to creatinine. Then, each spectrum was segmented from 0.6 to 9.4 ppm into consecutive bins of fixed size (0.0088 ppm). The pertaining regional integrals (bin intensities) were calculated, excluding the residual water regions (4.5–6.0) ppm. A bucket table was generated, wherein columns represented bin numbers and rows represented NMR sample numbers.

Principal component analysis (PCA): PCA is a standard unsupervised multivariate technique that consists of performing a coordinate transformation to try to separate relevant values from residual ones, e.g., noise. Ideally, it projects correlated variance distributed over several variables onto single new variables (i.e., the principal components), which simplify the visualization and interpretation. In this cohort, PCA was used for visualization and as a dimension reduction technique for further multivariate statistical analyses.

PCA/CA/k-NN classification: A classification approach different from SIMCA is needed if a sample needs to be classified with respect to multiple co-existing classes. Starting from a bucket table of a model set of samples, PCA is first applied for dimension reduction. Then, canonical analysis (CA) in combination with MANOVA is applied to determine the subspace for maximum class separation and its respective dimension. Finally, a classification rule is introduced, e.g., via the k-nearest neighbor (k-NN) concept. This produces the PCA/CA/k-NN classification procedure; for classification of a new test sample, the sample is projected into the PCA-CA subspace first, and k-NN is used to assign its class membership.

Monte Carlo embedded cross-validation (MCCV): PCA/CA/k-NN classification is a supervised method. Related models are established in a supervised manner, wherein the class membership of each object is known during the training phase. In order not to overfit any data, extensive validation is needed. We used the MCCV approach to maximize the rate of correct classification, and the confusion matrix has been obtained with an explained variance of 99%, 16 Monte Carlo runs (MC), an 8-fold cross-validation (CV).

### 4.7. Identification of Unknown Metabolites

To investigate unknown metabolites using NMR only, we further analyzed the samples using ultraperformance liquid chromatography–high-resolution mass spectrometry (UPLC-HR-MS). Details of the UHPLC-MS method are shown in Supplementary Material.

### 4.8. Statistical Analysis of Targeted Analysis

Metabolomic targeted statistics were performed with EXCEL, XLSTAT (2022.4.1) and IBM^®^ SPSS^®^ Statistics using the resultant data from PKU samples (*n* = 51) compared to age-matched healthy control samples (*n* = 51). A Mann–Whitney U-test and box plots were used to describe the 95th percentile confidence values and medians, respectively.

## Figures and Tables

**Table 1:** Data for age, gender, and urinary creatine for age-matched healthy controls compared to patients with PKU.

	Mean (±SD) or Percentage		*p*-Value
	Healthy Controls (*n* = 51)	PKU Patients (*n* = 51)	
Gender (% female)	52.9	51.0	
Age (years)	14.9 ± 9.2	14.9 ± 8.2	0.545
Creatinine [mmol/mol Crea]	8.2 ± 7.0	8.2 ± 7.3	0.857

**Table 2:** Blood Phe values, genetic alleles (when detected), sapropterin treatment status, dietary Phe intake, and genetic phenotype value (GPV) for patients with PKU (*n* = 51).

Patient ID	Phe Level (µmol/L Plasma)	Genetic Phenotype Value (GPV)	Phenotype	Allele 1	Allele 2	Sapropterin	Dietary Protein Intake (g/kg BW/day)
404	305	0	cPKU	IVS12 + 1G>A	p.R408W	no	0.420
397	289	0	cPKU	p.R408W	p.E280K	no	0.330
385	325		mPKU	IVS12 + 1G>A	p.F410I *	no	0.320
369	500	5.1	mPKU	IVS12 + 1G>A	p.Y414C	no	0.230
363	132		mPKU	IVS10−11G>A	Not detected	no	0.500
362	597	0	cPKU	p.R408W	p.R408W	no	0.240
359	467	0	cPKU	IVS10−11G>A	p.Y386C	no	0.300
356	287	5.1	mPKU	p.Y356X	p.Y414C	yes	1.101
405	429	0	cPKU	IVS12 + 1G>A	p.R408W	no	0.290
339	303	0	cPKU	IVS12 + 1G>A	p.R408W	no	0.440
347	279	0	cPKU	IVS12 + 1G>A	IVS10−11G>A	no	0.200
342	344	5.0	mPKU	p.R408W	p.D129Y	yes	1.700
336	135	0	cPKU	IVS12 + 1G>A	p.R408W	no	0.401
334	431	5.1	mPKU	p.G46S	p.Y414C	yes	2.700
335	475	5.1	mPKU	p.G46S	p.Y414C	yes	2.400
323	261	5.1	mPKU	p.F39L	p.Y414C	yes	1.102
316	528	5.1	mPKU	IVS12 + 1G>A	p.Y414C	no	0.390
314	546	0	cPKU	p.R408W	p.T266E	no	0.180
333	36	0	cPKU	R252W	p.R252W	no	0.100
313	254	5.1	mPKU	p.Y414C	p.W120X	yes	0.501
306	692	0	cPKU	p.R158Q	IVS12 + 1G>A	no	0.400
298	533	5.1	mPKU	p.R408W	p.Y414C	yes	0.320
302	513	0	cPKU	IVS10nt−11G>A	C.−473−?_168+?du	no	0.070
295	627	0	cPKU	IVS12 + 1G>A	P281L	no	0.070
292	434	0	cPKU	IVS12 + 1G>A	IVS12 + 1G>A	no	0.180
282	392	0	cPKU	IVS12 + 1G>A	IVS10−11G>A	no	0.200
278	622	6.9	mPKU	p.R408W	p.E390G	no	1.290
254	327	2.0	cPKU	p.L48S	IVS12 + 1G>A	yes	0.620
253	968	0	cPKU	IVS12 + 1G>A	E221D222duAG	no	0.110
258	682	0	cPKU	IVS12 + 1G>A	p.R408W	no	0.230
249	840	0	cPKU	IVS12 + 1G>A	p.R408W	no	0.250
236	1504	0	cPKU	IVS10−11G>A	p.Y386C	no	0.550
242	700	1.1	cPKU	IVS12 + 1G>A	p.I65T	yes	0.460
272	1010	0	cPKU	IVS10−11G>A	p.R408W	no	0.301
243	969	0	cPKU	IVS12 + 1G>A	p.R252W	no	0.502
237	277	0	cPKU	p.R408W	p.R408W	no	0.290
238	984	0	cPKU	IVS12 + 1G>A	IVS12 + 1G>A	no	0.120
235	1175		cPKU	IVS12 + 1G>A	Not detected	no	0.220
211	468	2.6	cPKU	p.R408W	p.A104D	yes	0.490
212	1163	0	cPKU	IVS10−11G>A	p.R408W	no	0.302
196	1292	0	cPKU	IVS12 + 1G>A	p.E221D	no	0.750
190	869	0	cPKU	p.P281L	p.R243X	no	1.103
191	766	0	cPKU	IVS12 + 1G>A	p.R408W	no	0.440
176	83	0	mPKU	IVS12 + 1G>A	p.R408W	no	0.180
168	831		cPKU	Not detected	Not detected	no	0.230
143	1590		cPKU	IVS12 + 1G>A	not detected	no	1.200
145	1344	2.6	cPKU	IVS1 + 5G>T	p.A104D	no	1.000
137	1118	5.1	mPKU	IVS12 + 1G>A	p.Y414C	no	1.100
129	1613		cPKU	IVS12 + 1G>A	unclear	no	0.600
126	1955	0	cPKU	IVS12 + 1G>A	p.D282N	no	0.800
121	1390	0	cPKU	IVS12 + 1G>A	p.R158Q	no	1.104

* The blood Phe value for patient ID 385 was borderline HPA and mPKU described in [26]. BW: bodyweight.

**Table 3:** A total of 24 urinary metabolites that showed a significant difference (*p* < 0.05) from healthy age-matched controls (*n* = 51). They are ranked according to their fold change. They belong to very different substance classes, pathways, and origins (s. text). * mmol/mol creatinine, ** *N*-methyl-2-pyridone-5-carboxamide, *** *N*1-Methyl-4-pyridone-3-carboxamide, HEA = healthy controls, PKU = phenylketonuria, SD = standard deviation.

	HEA			PKU				
	n	Mean *	SD	n	Mean *	SD	*p*-Value	Fold Change
**Phenylpyruvic acid**	**42**	10.8	10.2	**46**	73.9	126.6	**0.003**	**6.853**
**D-Mandelic acid**	**10**	2.1	2.2	**8**	9.9	8.2	**0.013**	**4.762**
**2-Furoylglycine**	**21**	12.7	17.7	**20**	37.5	39.8	**0.007**	**2.950**
**Tartaric acid**	**51**	10.3	24.2	**51**	27.3	26.9	**0.0001**	**2.665**
**Phenylacetic acid**	**42**	5.5	5.6	**38**	13.9	14.7	**0.003**	**2.558**
**Glycine**	**51**	117.8	124.1	**51**	282.6	342.2	**0.008**	**2.400**
**Methionine**	**13**	3.6	1.4	**12**	8.4	8.6	**0.034**	**2.343**
**Acetic acid**	**51**	8.9	7.2	**51**	17.2	13.3	**0.001**	**1.931**
**Phenylalanine**	**42**	21.0	18.3	**47**	39.7	21.4	**0.0001**	**1.889**
**Neopterin**	**51**	3.0	5.3	**51**	5.2	7.6	**0.022**	**1.750**
2PY ** and 4PY ***	**51**	5.9	3.5	**51**	10.1	5.3	**0.0001**	**1.728**
**L-Citramalic acid**	**51**	18.5	12.0	**51**	31.8	24.9	**0.004**	**1.721**
**Maleic acid**	**50**	1.1	1.9	**51**	1.7	2.2	**0.0001**	**1.639**
**Adenine**	**45**	2.0	2.0	**50**	3.2	3.7	**0.03**	**1.570**
**1-Methylnicotinamide**	**51**	9.4	9.0	**51**	13.6	16.0	**0.027**	**1.459**
**2-Hydroxyisovaleric acid**	**36**	1.2	0.7	**31**	1.7	1.1	**0.01**	**1.459**
**Oxaloacetic acid**	**50**	27.8	26.9	**47**	38.8	25.1	**0.008**	**1.396**
**3-Methylglutaconic acid**	**51**	5.6	2.9	**51**	7.8	4.4	**0.003**	**1.392**
**Valine**	**51**	4.6	2.9	**51**	6.4	5.2	**0.011**	**1.392**
**Acetoacetic acid**	**46**	11.8	9.9	**48**	16.1	8.5	**0.006**	**1.371**
**Guanidinoacetic acid**	**51**	93.1	71.5	**50**	74.1	67.7	**0.026**	**0.796**
**Creatine**	**49**	201.4	370.1	**49**	160.2	269.7	**0.031**	**0.796**
**Dimethylamine**	**51**	40.2	27.2	**51**	30.3	20.6	**0.041**	**0.753**
**Allantoin**	**51**	16.2	11.3	**48**	7.1	5.9	**0.0001**	**0.440**

## Data Availability

Data are available on request from gefde@gmx.de.

## Supplementary Materials

The following supporting information can be downloaded at: https://www.mdpi.com/article/10.3390/molecules28134916/s1, Figure S1: UPLC chromatogram and NMR revealing two metabolites. Figure S2: Summary of statistical analysis for 24 metabolites of age-matched healthy controls and PKU patients. Figure S3: Regression analysis of 24 metabolites with age in age-matched healthy controls and PKU patients. Figure S4: Regression analysis between metabolites in urine and actual Phe level in plasma and natural protein intake. Figure S5: Multifactorial analysis of metabolites using standard coefficient of variation. Table S1: List of metabolites as measured by the ex vivo 1H-NMR analysis in urine. Supplemental text: Method for identification of unknown metabolites with UPLC-MS.
