# Supplementary material for: Phenylketonuria (PKU) Urinary Metabolomic Phenotype Is Defined by Genotype and Metabolite Imbalance: Results in 51 Early Treated Patients Using Ex Vivo 1H-NMR Analysis
Source: Molecules. 2023 Jun 22;28(13):4916. doi: 10.3390/molecules28134916 (PMC10343293; doi:10.3390/molecules28134916)
Supplement: Supplementary file 1 [file molecules-28-04916-s001.zip › molecules-2390382-supplementary.pdf]

Supplementary Materials

Phenylketonuria (PKU) Urinary Metabolomic Phenotype Is Defined by Genotype and Metabolite Imbalance: Results in 51 Early Treated Patients Using Ex Vivo ^{1}H-NMR Analysis

Claire Cannet [1], Allan Bayat [2], Georg Frauendienst-Egger [3], Peter Freisinger [3], Manfred Spraul [1], Nastassja Himmelreich [4], Musa Kockaya [5], Kirsten Ahring [2], Markus Godejohann [1], Anita MacDonald [6] and Friedrich Trefz [7,*]

[1] Bruker Biospin, 76275 Ettlingen, Germany; claire.cannet@bruker.com (C.C.)
[2] Kennedy Centre, Center for PKU, 2600 Glostrup, Denmark
[3] Department of Pediatrics, School of Medicine, University of Tübingen, 72074 Tübingen, Germany
[4] CEGAT, Human Genetic Institute, 72076 Tübingen, Germany
[5] Private Pediatric Practice, 68307 Mannheim, Germany
[6] Dietetic Department, Birmingham Children's Hospital, Birmingham B4 6NH, UK
[7] Metabolic Consulting Reutlingen, 72766 Reutlingen, Germany
* Correspondence: friedrich.trefz@metabolic-consulting.de

Supplementary Table S1. List of metabolites as measured by the ex vivo ^{1}H-NMR analysis in urine.

NMR-Metabolites (n=149)					
1	Creatinine	51	Leucine	101	2-Hydroxyphenylacetic acid
2	1-Methylguanidine	52	4-Pyridoxic acid	102	Benzoic acid
3	Dimethylamine	53	Adenine	103	L-Pyroglutamic acid
4	Alanine	54	Acetoacetic acid	104	2-Methylsuccinic acid
5	Betaine	55	Thymol	105	Myo-Inositol
6	Glycine	56	L-Fucose	106	Glutamine
7	Guanidinoacetic acid	57	Propylene glycol	107	DL-Alloisoleucine
8	N,N-Dimethylglycine	58	L-Tryptophan	108	N-Acetylglutamate
9	N-Acetylaspartic acid	59	Uracil	109	D-Galactonic acid
10	Valine	60	Phenylalanine	110	4-Hydroxyphenyllactic acid
11	Syringic acid	61	Phenylpyruvic acid	111	Paracetamol
12	Acetic acid	62	Ethylmalonic acid	112	Succinylacetone
13	Citric acid	63	Xanthurenic acid	113	Galactitol
14	Formic acid	64	Phenylacetic acid	114	Glycerol
15	Fumaric acid	65	Glutaric acid	115	Isopropanol
16	Lactic acid	66	Propionic acid	116	3-Methylcrotonylglycine
17	Succinic acid	67	Citrulline	117	Cystine
18	Tartaric acid	68	2-Oxoglutaric acid	118	3-Hydroxyvaleric acid
19	Pantothenic acid	69	Sarcosine	119	L-Threonic acid
20	3-Hydroxyisovaleric acid	70	3-Hydroxyglutaric acid	120	Tyramine
21	3-Methylglutaconic acid	71	3-Phenyllactic acid	121	N-Acetylphenylalanine
22	L-Citramalic acid	72	4-Hydroxyphenylacetic acid	122	2-Oxoisovaleric acid
23	Glycolic acid	73	L-Isoleucine	123	Ethanol
24	Acetone	74	2-Hydroxyisovaleric acid	124	Propionylglycine
25	Pyruvic acid	75	Uridine	125	5-Aminolevulinic acid
26	1-Methylhydantoin	76	3-Methyl-2-oxovaleric acid	126	Paracetamol-glucuronide
27	1-Methylnicotinamide	77	N-Isovaleroylglycine	127	2-Hydroxy-4-methylvaleric acid
28	2PY plus 4PY	78	Imidazole	128	2-Ketobutyric acid
29	Caffeine	79	D-Galactose	129	Argininosuccinic acid
30	Inosine	80	3-Hydroxybutyric acid	130	Glutamic acid
31	Neopterin	81	Methylmalonic acid	131	N-Acetyltyrosine
32	Orotic acid	82	2-Furoylglycine	132	4-Ethylphenol
33	Oxypurinol	83	4-Aminobutyric acid	133	D-Panthenol
34	Theobromine	84	2-Oxoisocaproic acid	134	5-Aminopentanoic acid
35	D-Glucose	85	Thymine	135	Isobutyrylglycine
36	D-Lactose	86	3-Hydroxypropionic acid	136	L-Carnosine
37	Methanol	87	Choline	137	L-Homocystine
38	Trimethylamine	88	Cytosine	138	4-Aminohippuric acid
39	Arginine	89	D-Gluconic acid	139	Pyrocatechol
40	Taurine	90	Methionine	140	E-Glutaconic acid
41	Hippuric acid	91	4-Hydroxyhippuric acid	141	L-Ascorbic acid
42	Maleic acid	92	Butyric acid	142	Citraconic acid
43	Trigonelline	93	D-Mannose	143	Pimelic acid
44	Oxaloacetic acid	94	Tiglylglycine	144	Malic acid
45	1,3-Dimethyluric acid	95	1-Methylhistidine	145	Acetoine
46	Proline betaine	96	Dihydrouracil	146	DL-Kynurenin
47	Allantoin	97	4-Hydroxyphenylpyruvic acid	147	1-Methyladenosine
48	Dihydrothymine	98	D-Mandelic acid	148	Adenosine
49	Creatine	99	D-Mannitol	149	Quinolinic acid
50	3-Hydroxy-3-methylglutaric acid	100	3-Aminoisobutyric acid		

Supplementary Figure S1. UPLC chromatogram (**A**) and NMR (**B**) revealing two metabolites (quantified as “Allopurinol”): Peak #1: *N*-methyl-2-pyridone-5-carboxamide and Peak #2: *N*1-Methyl-4-pyridone-5-carboxamide using Heteronuclear Multiple Bond Correlation.

A

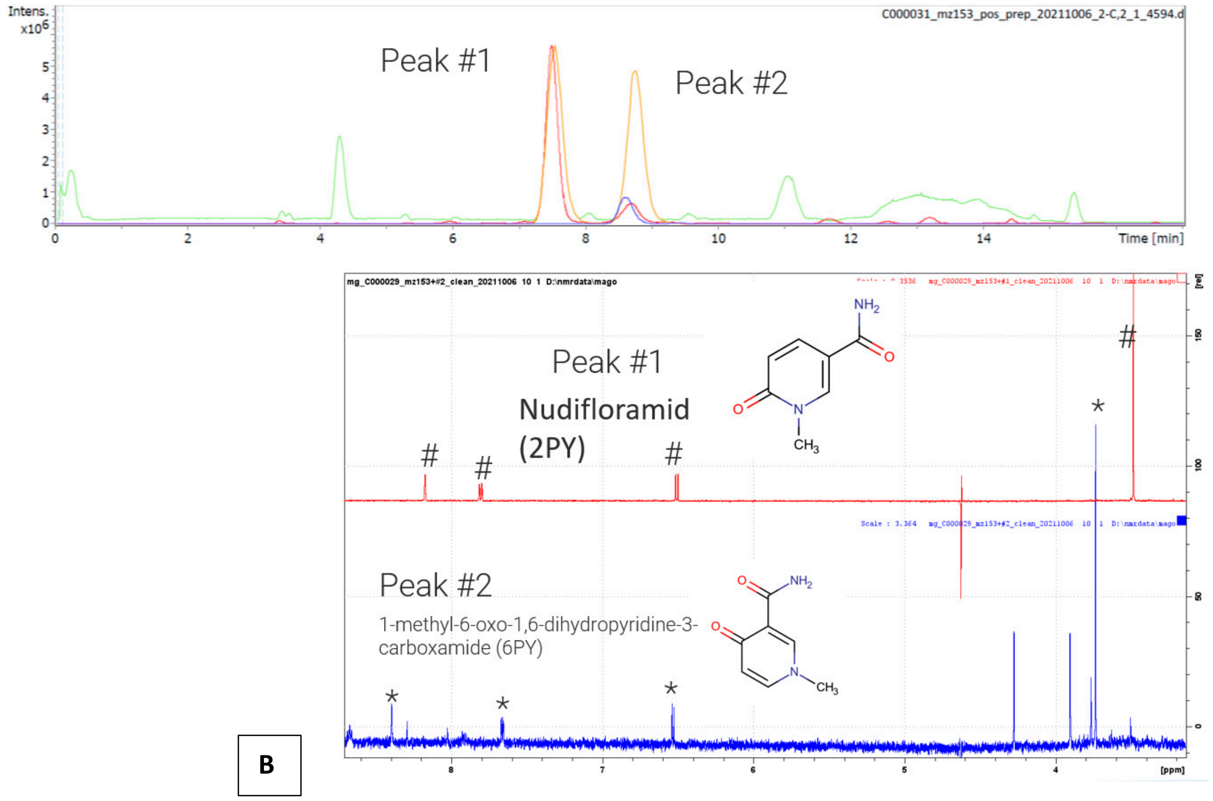

B

Supplementary Figure S2. Summary of statistical analysis for 24 metabolites of age-matched healthy controls (HEA; *n*=51) and patients with phenylketonuria (PKU; *n*=51). Mann Whitney U-test, box blot showing median and 95th percentile values in urine (mmol/mol creatinine).

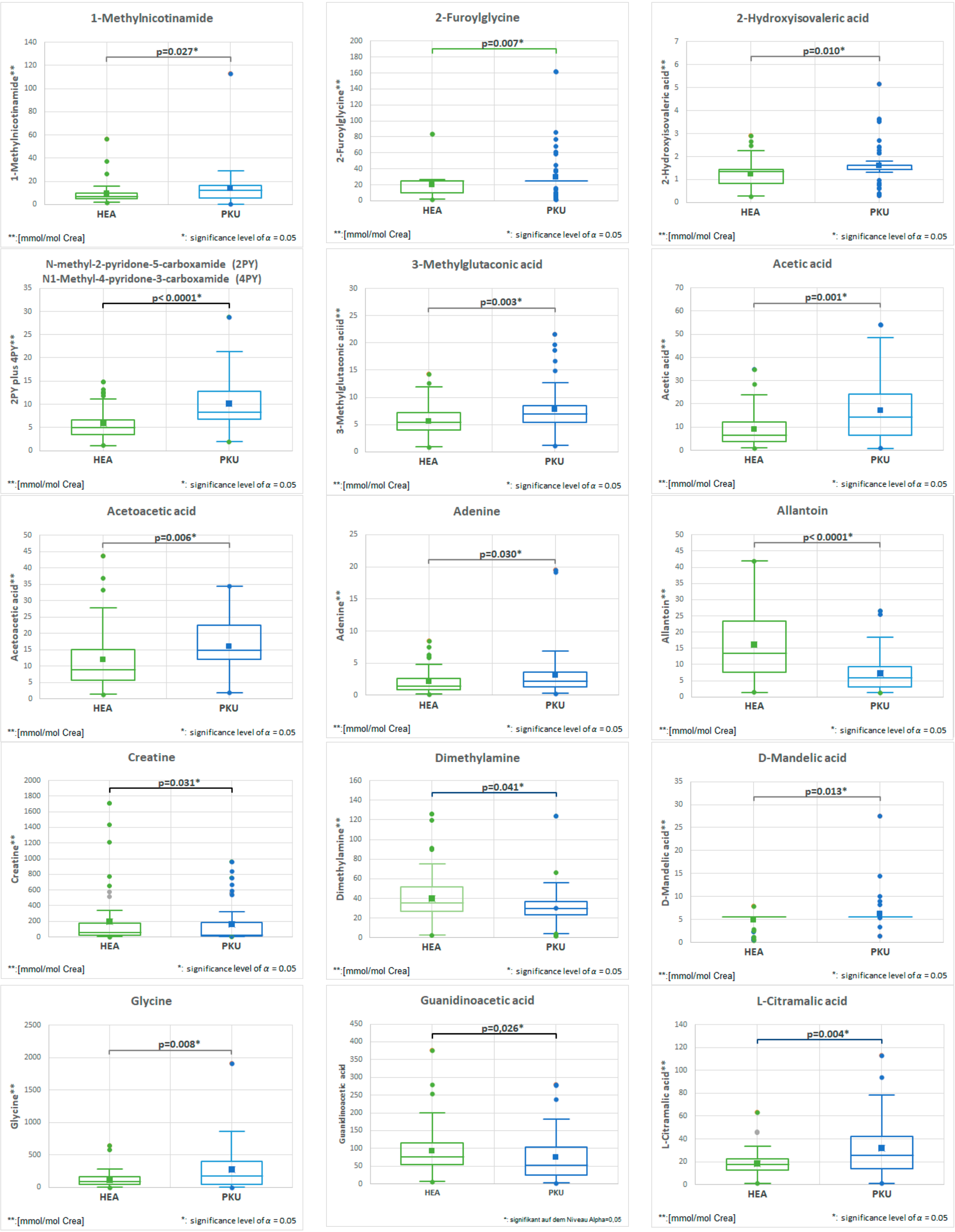

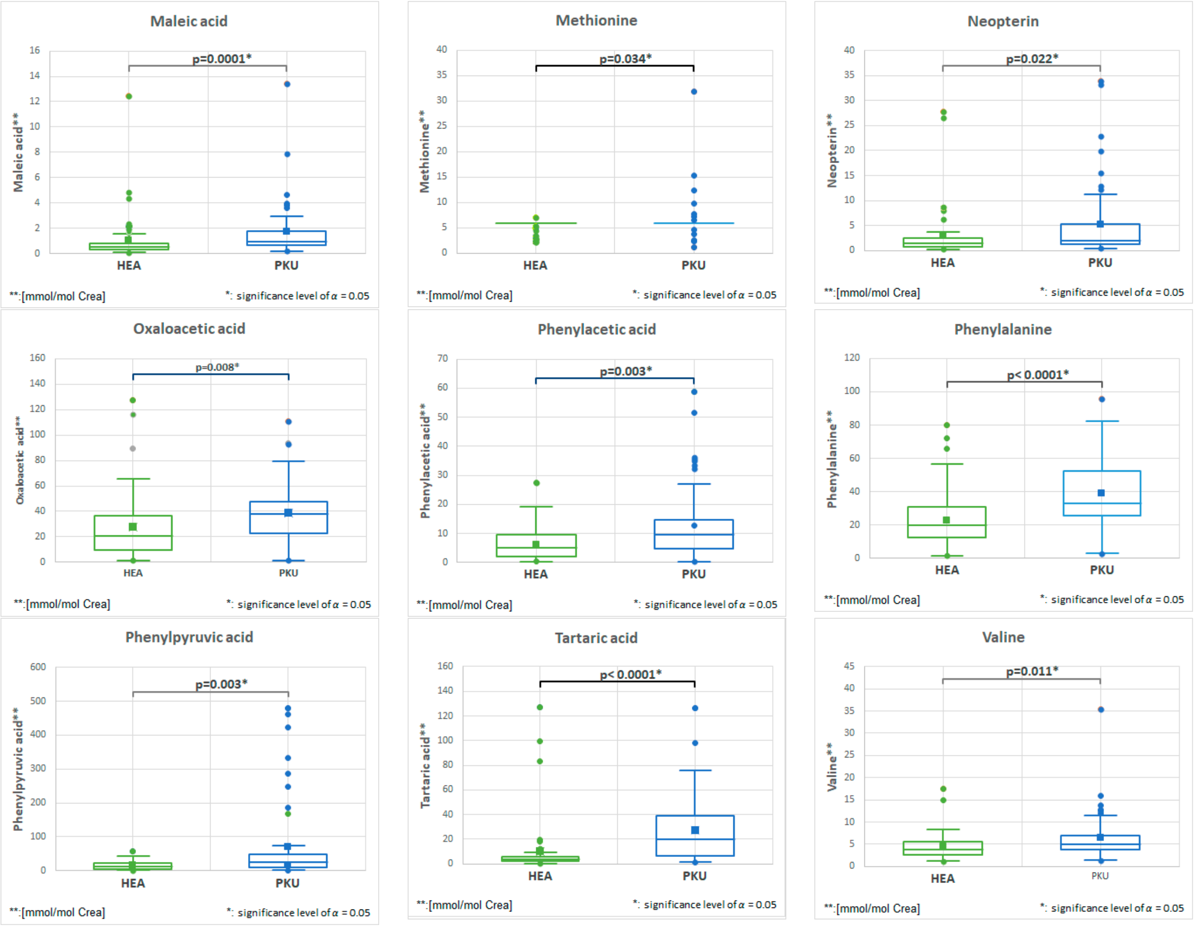
Maleic acid
p=0.0001*
Maleic acid**
HEA
PKU
**:[mmol/mol Crea]
*: significance level of α = 0.05
Methionine
p=0.034*
Methionine**
HEA
PKU
**:[mmol/mol Crea]
*: significance level of α = 0.05
Neopterin
p=0.022*
Neopterin**
HEA
PKU
**:[mmol/mol Crea]
*: significance level of α = 0.05
Oxaloacetic acid
p=0.008*
Oxaloacetic acid**
HEA
PKU
**:[mmol/mol Crea]
*: significance level of α = 0.05
Phenylacetic acid
p=0.003*
Phenylacetic acid**
HEA
PKU
**:[mmol/mol Crea]
*: significance level of α = 0.05
Phenylalanine
p< 0.0001*
Phenylalanine**
HEA
PKU
**:[mmol/mol Crea]
*: significance level of α = 0.05
Phenylpyruvic acid
p=0.003*
Phenylpyruvic acid**
HEA
PKU
**:[mmol/mol Crea]
*: significance level of α = 0.05
Tartaric acid
p< 0.0001*
Tartaric acid**
HEA
PKU
**:[mmol/mol Crea]
*: significance level of α = 0.05
Valine
p=0.011*
Valine**
HEA
PKU
**:[mmol/mol Crea]
*: significance level of α = 0.05

Supplementary Figure S3. Regression analysis of 24 metabolites with age in age-matched healthy controls (HEA, red; *n*=51) and patients with phenylketonuria (PKU, blue; *n*=51). Significant differences between groups (HEA/PKU) and regression "Age" are shown in red.

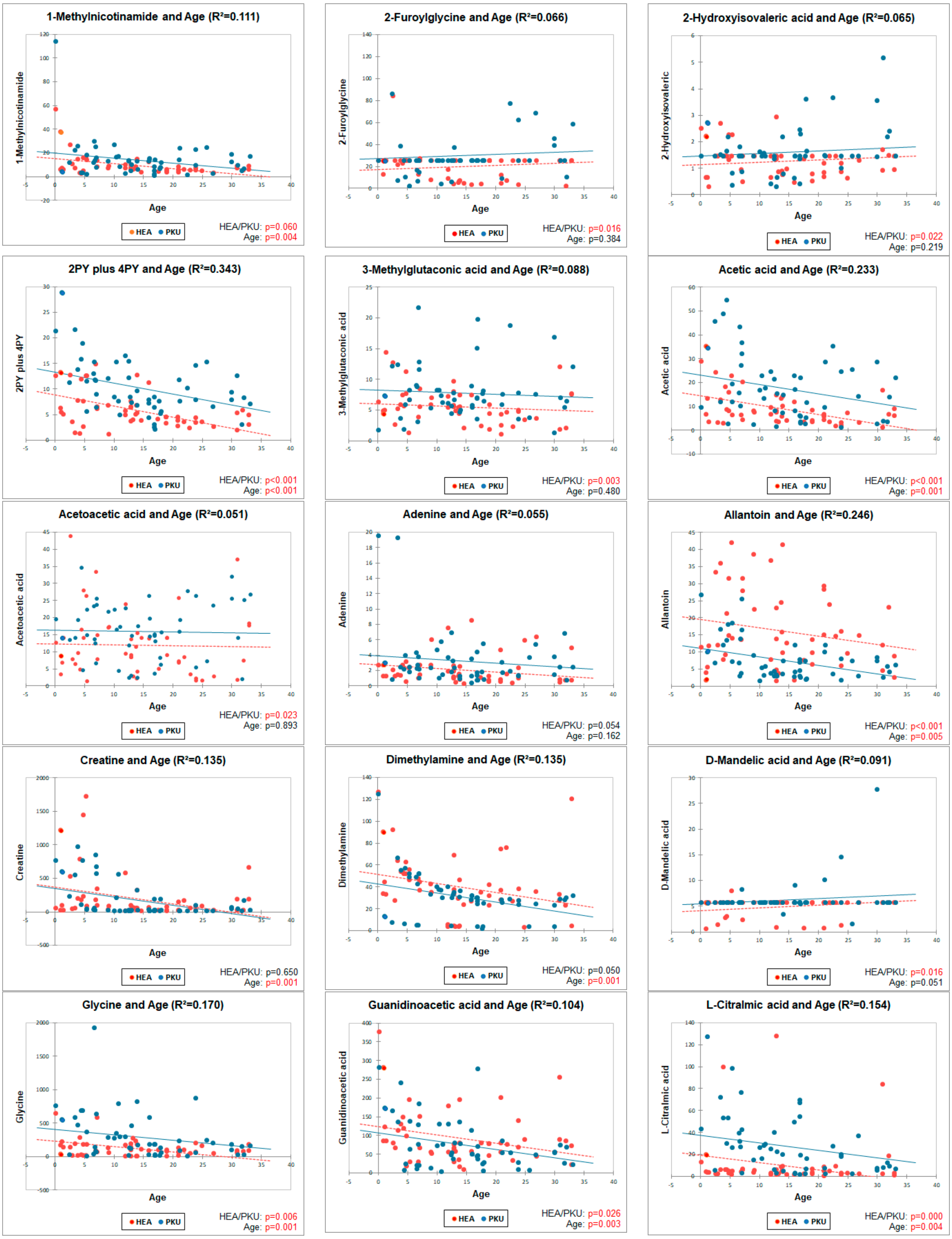

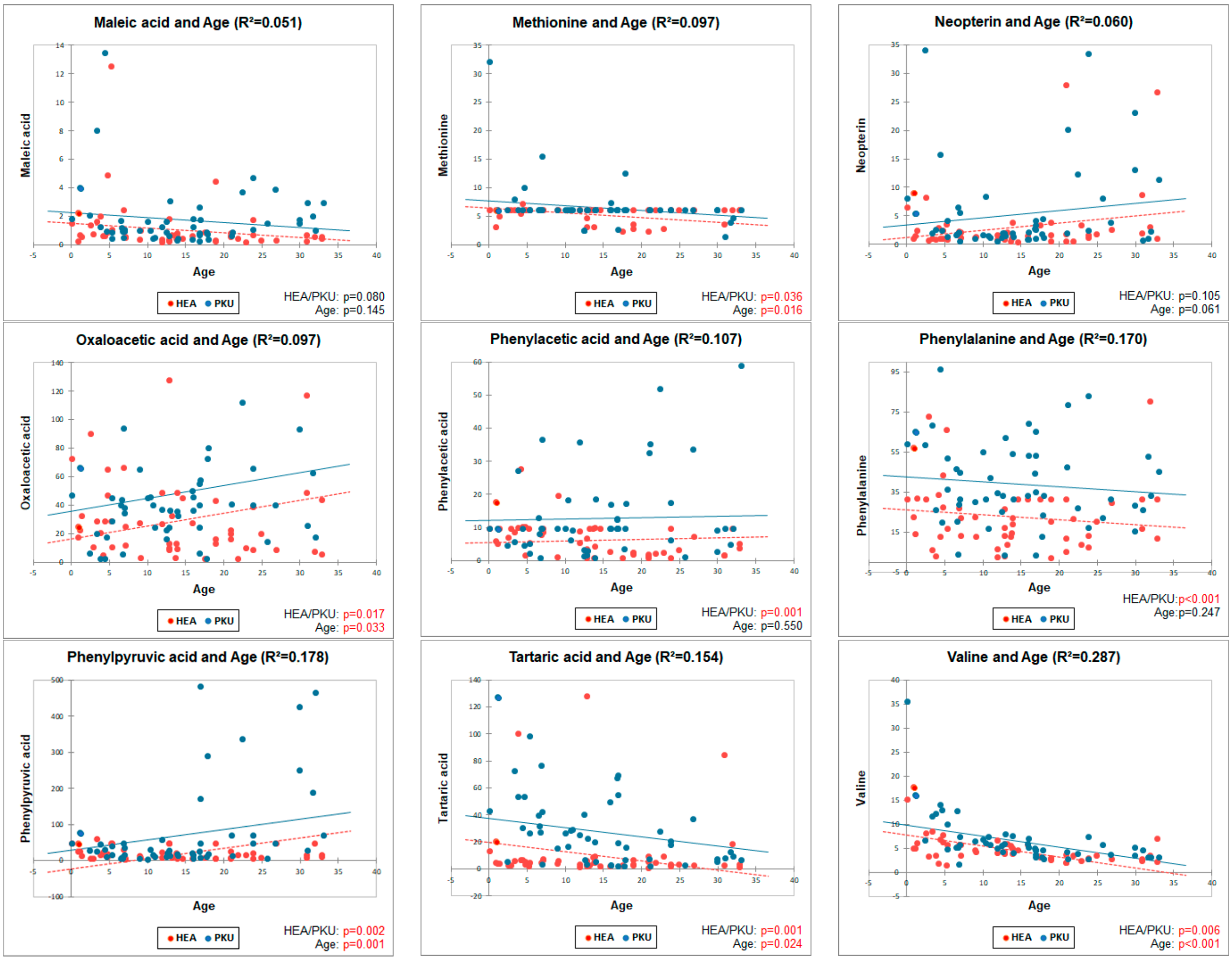
Maleic acid and Age (R²=0.051)
Maleic acid
Age
HEA
PKU
HEA/PKU: p=0.080
Age: p=0.145
Methionine and Age (R²=0.097)
Methionine
Age
HEA
PKU
HEA/PKU: p=0.036
Age: p=0.016
Neopterin and Age (R²=0.060)
Neopterin
Age
HEA
PKU
HEA/PKU: p=0.105
Age: p=0.061
Oxaloacetic acid and Age (R²=0.097)
Oxaloacetic acid
Age
HEA
PKU
HEA/PKU: p=0.017
Age: p=0.033
Phenylacetic acid and Age (R²=0.107)
Phenylacetic acid
Age
HEA
PKU
HEA/PKU: p=0.001
Age: p=0.550
Phenylalanine and Age (R²=0.170)
Phenylalanine
Age
HEA
PKU
HEA/PKU:p<0.001
Age:p=0.247
Phenylpyruvic acid and Age (R²=0.178)
Phenylpyruvic acid
Age
HEA
PKU
HEA/PKU: p=0.002
Age: p=0.001
Tartaric acid and Age (R²=0.154)
Tartaric acid
Age
HEA
PKU
HEA/PKU: p=0.001
Age: p=0.024
Valine and Age (R²=0.287)
Valine
Age
HEA
PKU
HEA/PKU: p=0.006
Age: p<0.001

Supplementary Figure S4. Regression analysis between metabolites in urine (mmol/mol creatinine) and actual phenylalanine level in plasma (μmol/l) and natural protein intake (g/kg bodyweight/day). Significant regression in red (p<0.05).

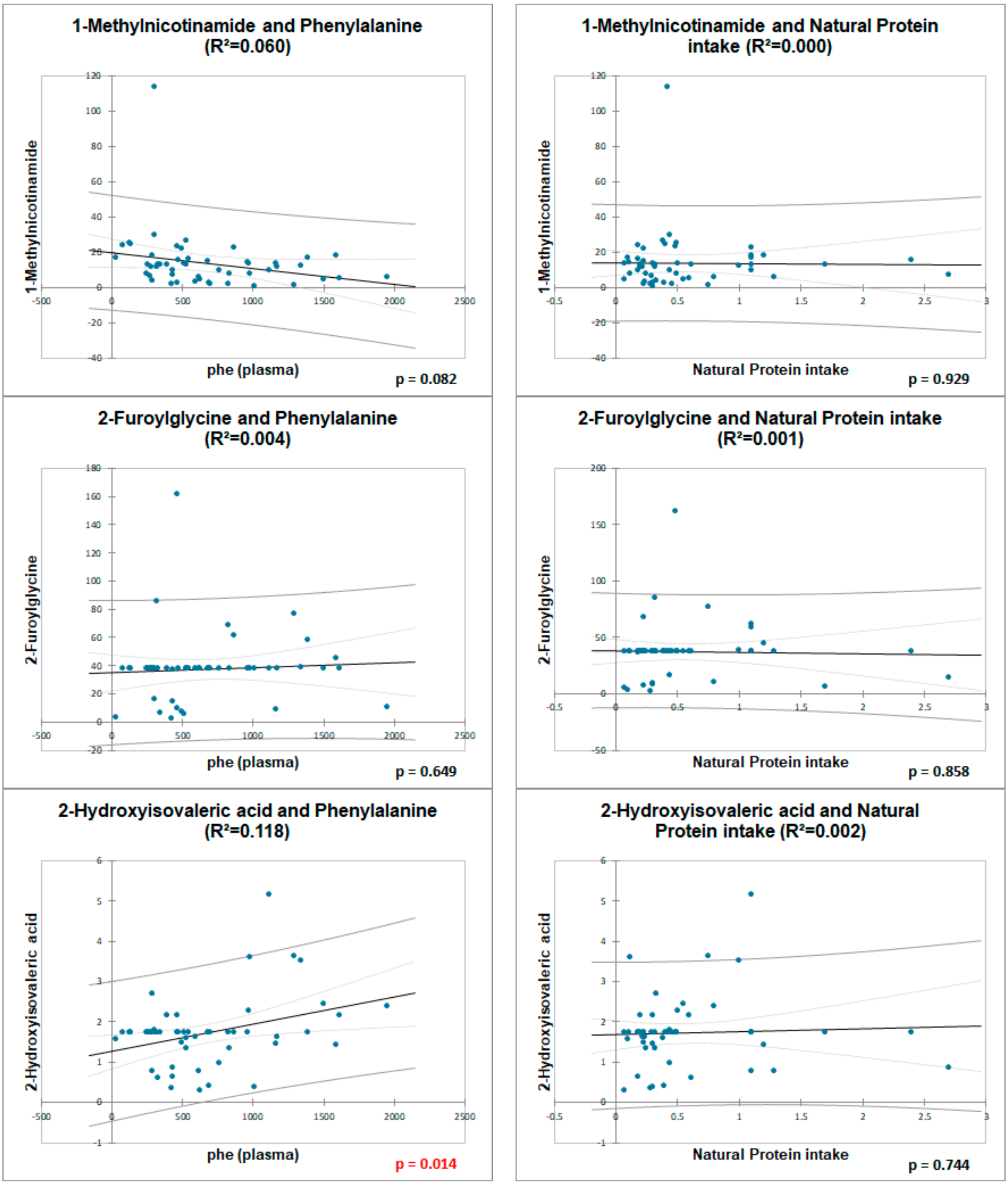

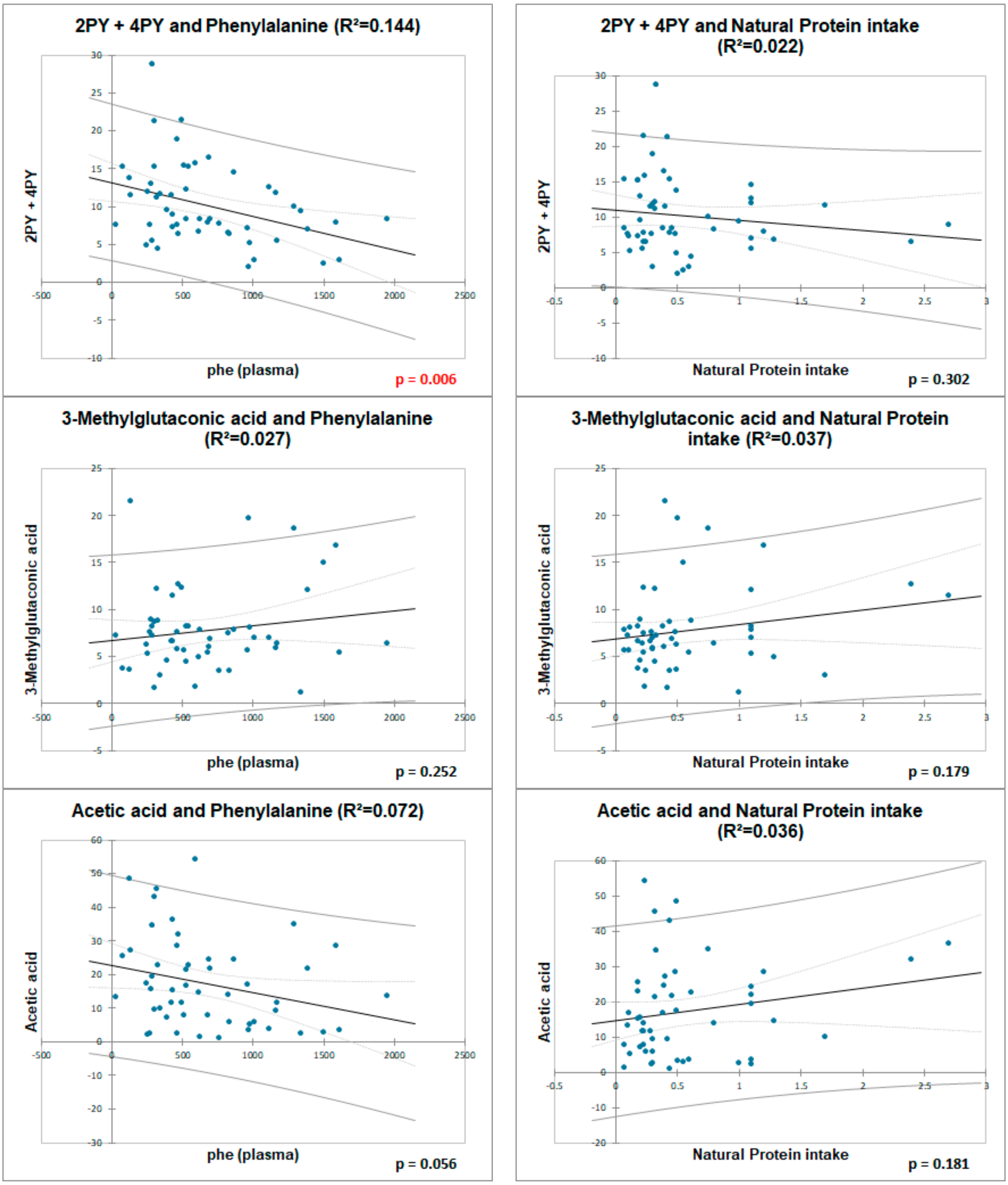
2PY + 4PY and Phenylalanine (R²=0.144)
2PY + 4PY
phe (plasma)
p = 0.006
2PY + 4PY and Natural Protein intake (R²=0.022)
2PY + 4PY
Natural Protein intake
p = 0.302
3-Methylglutaconic acid and Phenylalanine (R²=0.027)
3-Methylglutaconic acid
phe (plasma)
p = 0.252
3-Methylglutaconic acid and Natural Protein intake (R²=0.037)
3-Methylglutaconic acid
Natural Protein intake
p = 0.179
Acetic acid and Phenylalanine (R²=0.072)
Acetic acid
phe (plasma)
p = 0.056
Acetic acid and Natural Protein intake (R²=0.036)
Acetic acid
Natural Protein intake
p = 0.181

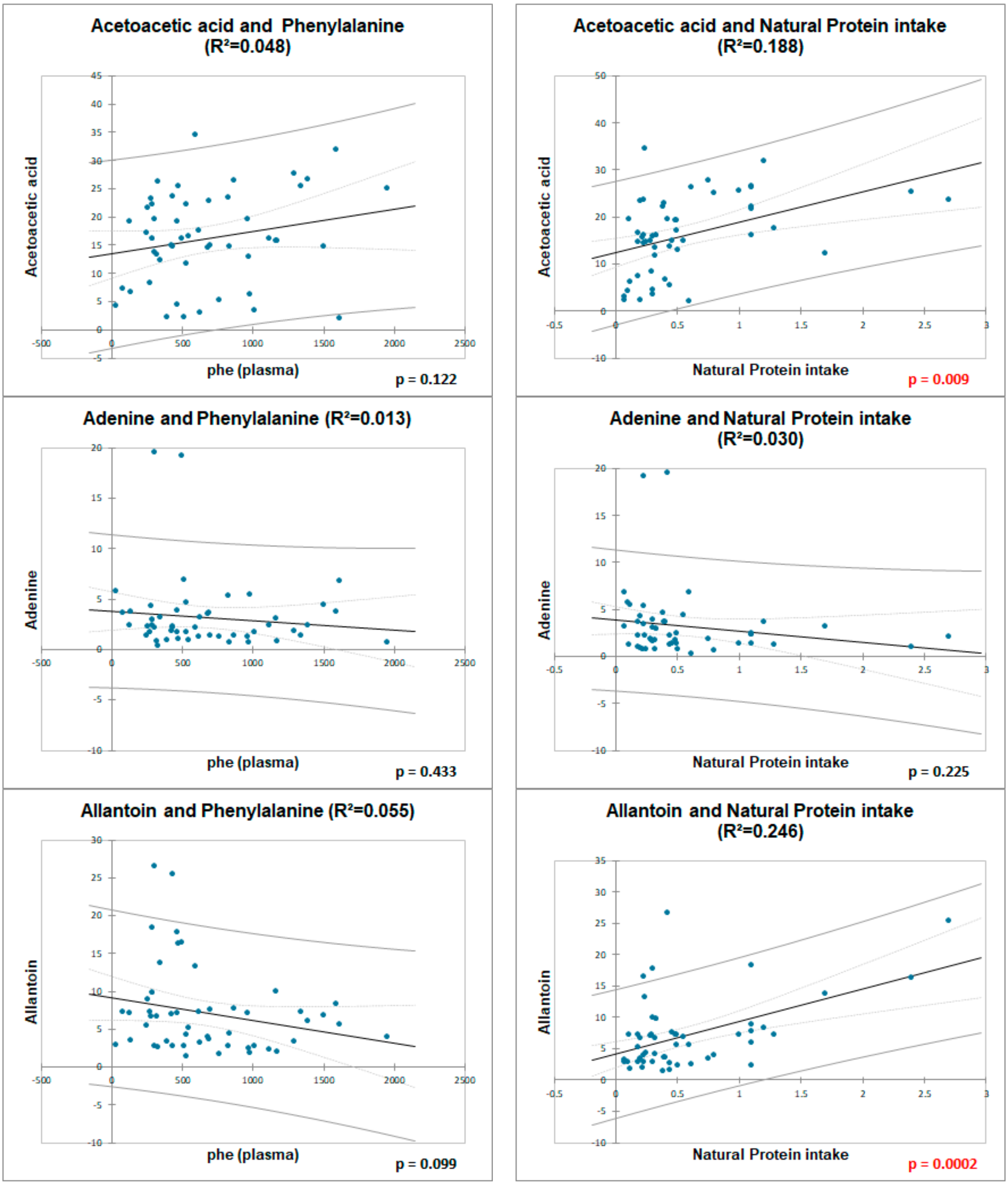
Acetoacetic acid and Phenylalanine
(R²=0.048)
Acetoacetic acid
phe (plasma)
p = 0.122
Acetoacetic acid and Natural Protein intake
(R²=0.188)
Acetoacetic acid
Natural Protein intake
p = 0.009
Adenine and Phenylalanine (R²=0.013)
Adenine
phe (plasma)
p = 0.433
Adenine and Natural Protein intake
(R²=0.030)
Adenine
Natural Protein intake
p = 0.225
Allantoin and Phenylalanine (R²=0.055)
Allantoin
phe (plasma)
p = 0.099
Allantoin and Natural Protein intake
(R²=0.246)
Allantoin
Natural Protein intake
p = 0.0002

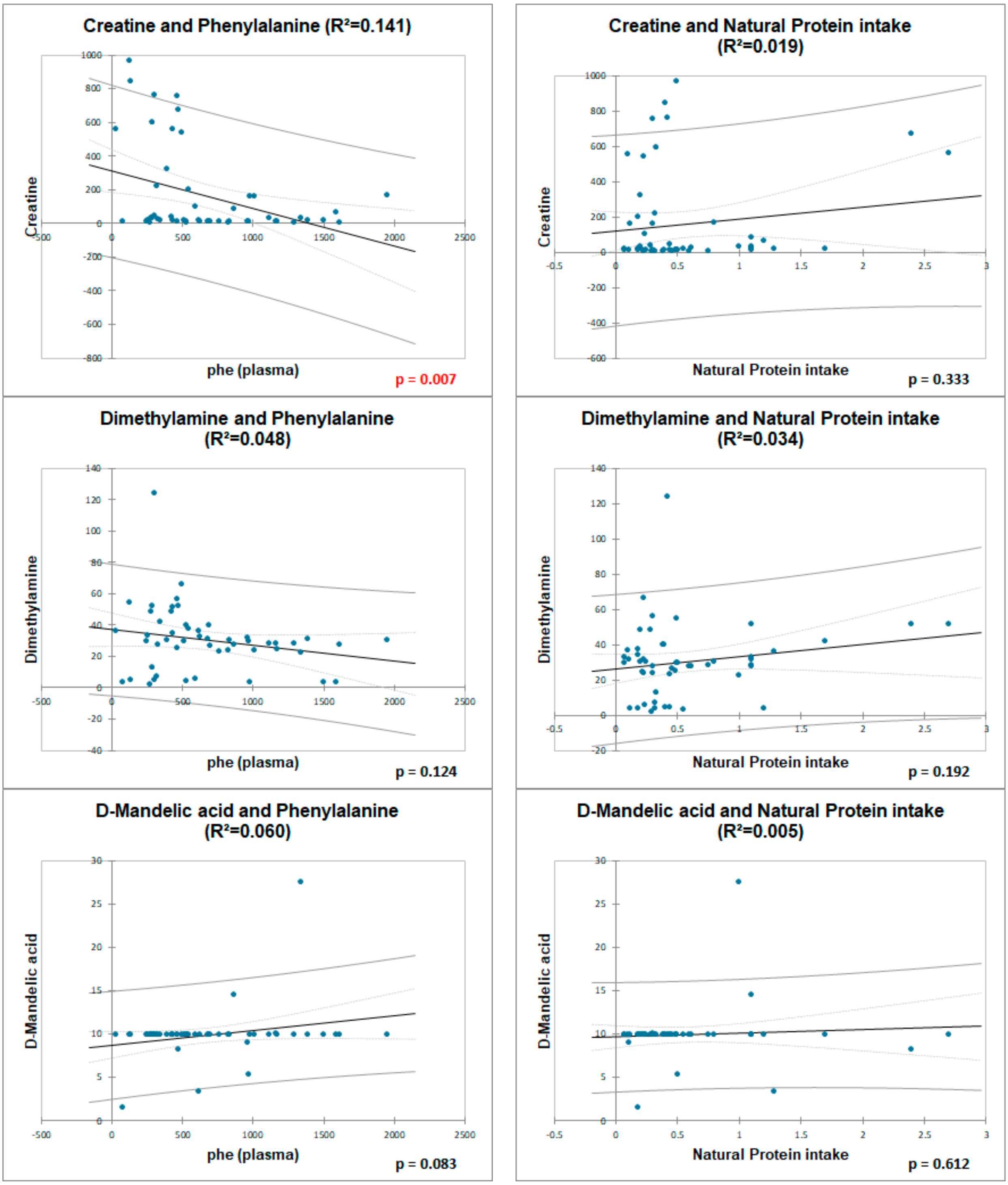
Creatine and Phenylalanine (R²=0.141)
Creatine
phe (plasma)
p = 0.007
Creatine and Natural Protein intake (R²=0.019)
Creatine
Natural Protein intake
p = 0.333
Dimethylamine and Phenylalanine (R²=0.048)
Dimethylamine
phe (plasma)
p = 0.124
Dimethylamine and Natural Protein intake (R²=0.034)
Dimethylamine
Natural Protein intake
p = 0.192
D-Mandelic acid and Phenylalanine (R²=0.060)
D-Mandelic acid
phe (plasma)
p = 0.083
D-Mandelic acid and Natural Protein intake (R²=0.005)
D-Mandelic acid
Natural Protein intake
p = 0.612

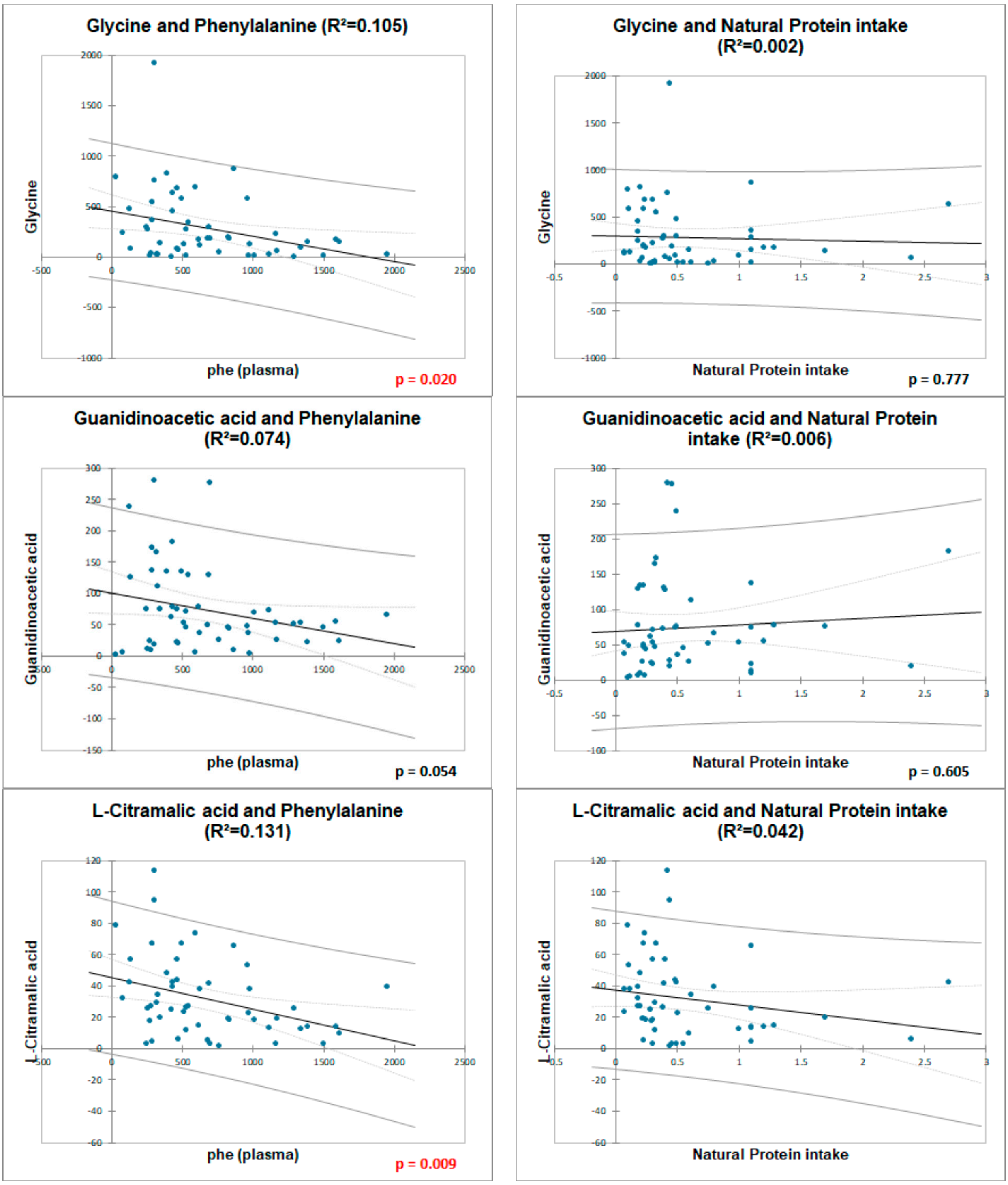

Glycine and Phenylalanine (R²=0.105)
Glycine
phe (plasma)
p = 0.020
Glycine and Natural Protein intake (R²=0.002)
Glycine
Natural Protein intake
p = 0.777
Guanidinoacetic acid and Phenylalanine (R²=0.074)
Guanidinoacetic acid
phe (plasma)
p = 0.054
Guanidinoacetic acid and Natural Protein intake (R²=0.006)
Guanidinoacetic acid
Natural Protein intake
p = 0.605
L-Citramalic acid and Phenylalanine (R²=0.131)
L-Citramalic acid
phe (plasma)
p = 0.009
L-Citramalic acid and Natural Protein intake (R²=0.042)
L-Citramalic acid
Natural Protein intake

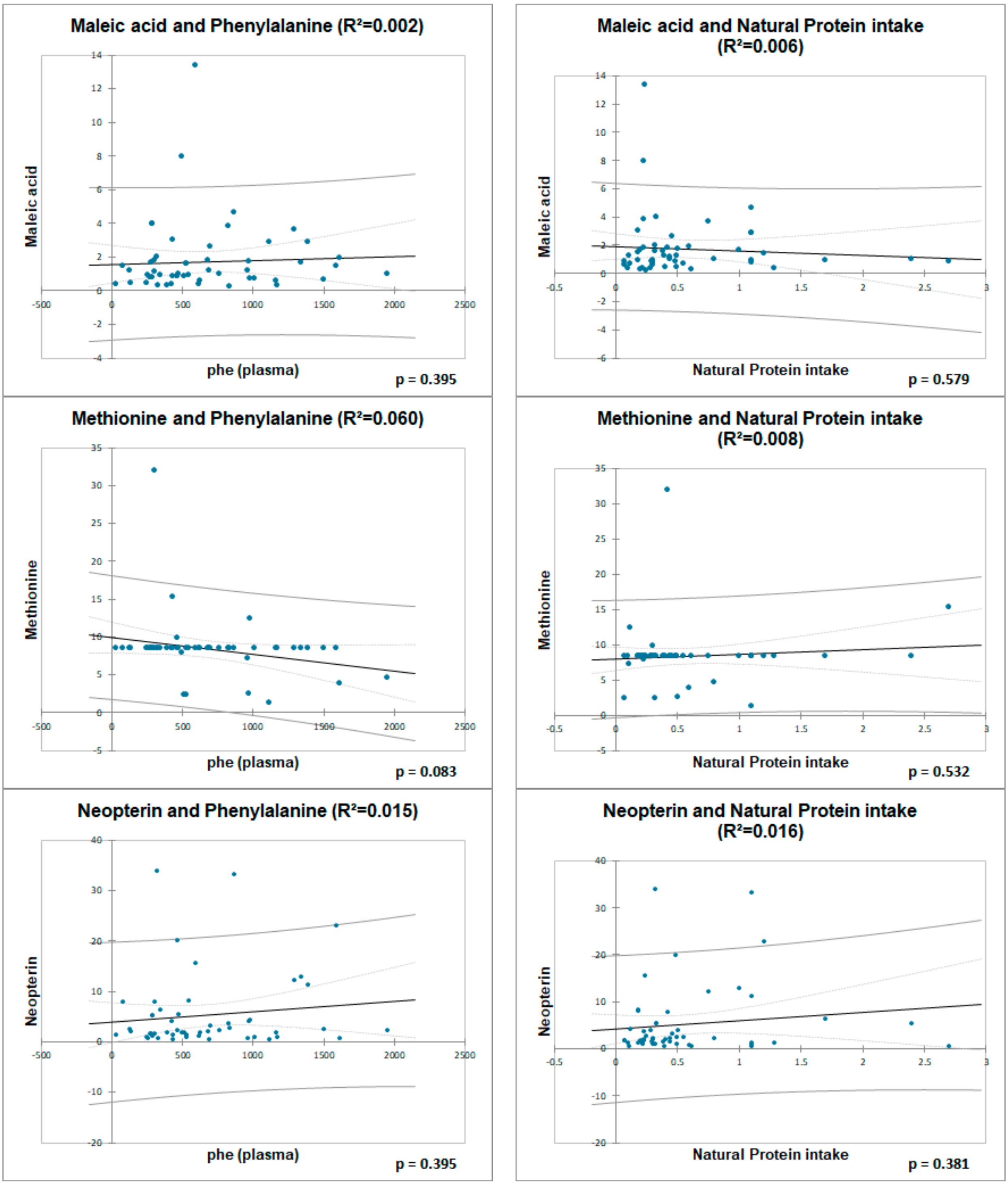
Maleic acid and Phenylalanine (R²=0.002)
Maleic acid
phe (plasma)
p = 0.395
Maleic acid and Natural Protein intake (R²=0.006)
Maleic acid
Natural Protein intake
p = 0.579
Methionine and Phenylalanine (R²=0.060)
Methionine
phe (plasma)
p = 0.083
Methionine and Natural Protein intake (R²=0.008)
Methionine
Natural Protein intake
p = 0.532
Neopterin and Phenylalanine (R²=0.015)
Neopterin
phe (plasma)
p = 0.395
Neopterin and Natural Protein intake (R²=0.016)
Neopterin
Natural Protein intake
p = 0.381

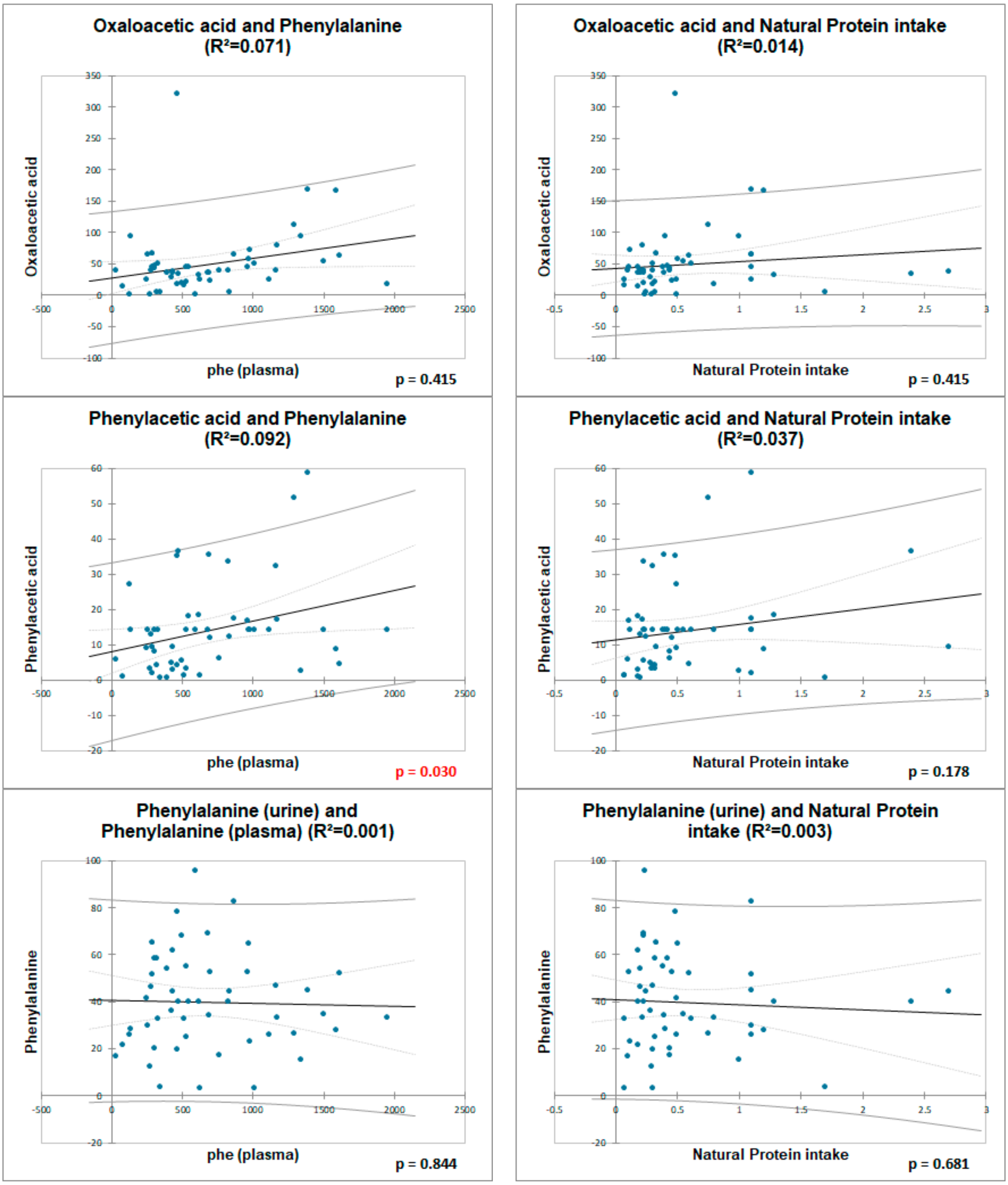
Oxaloacetic acid and Phenylalanine
(R²=0.071)
Oxaloacetic acid
phe (plasma)
p = 0.415
Oxaloacetic acid and Natural Protein intake
(R²=0.014)
Oxaloacetic acid
Natural Protein intake
p = 0.415
Phenylacetic acid and Phenylalanine
(R²=0.092)
Phenylacetic acid
phe (plasma)
p = 0.030
Phenylacetic acid and Natural Protein intake
(R²=0.037)
Phenylacetic acid
Natural Protein intake
p = 0.178
Phenylalanine (urine) and
Phenylalanine (plasma) (R²=0.001)
Phenylalanine
phe (plasma)
p = 0.844
Phenylalanine (urine) and Natural Protein
intake (R²=0.003)
Phenylalanine
Natural Protein intake
p = 0.681

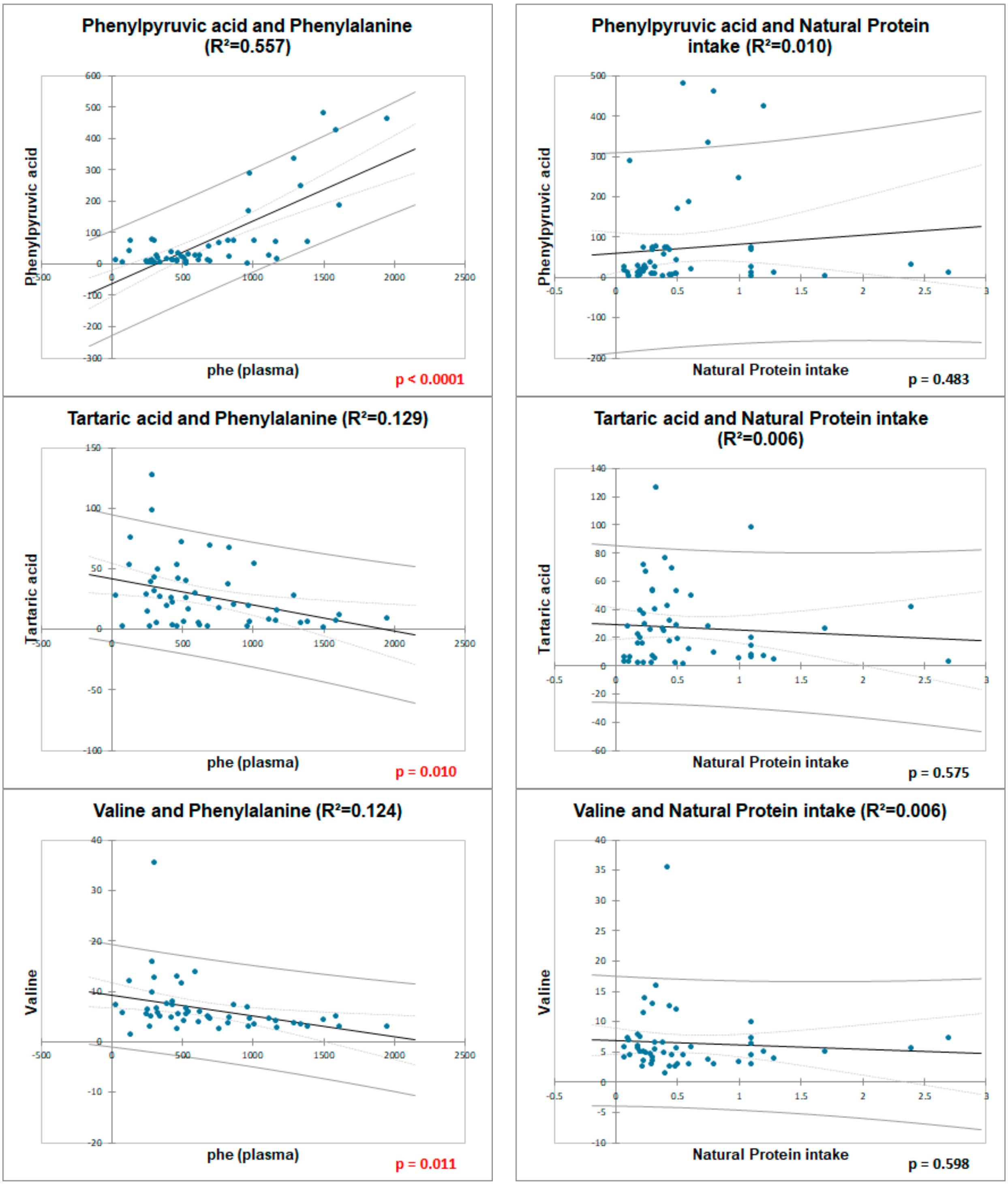
Phenylpyruvic acid and Phenylalanine (R²=0.557)
Phenylpyruvic acid
phe (plasma)
p < 0.0001
Phenylpyruvic acid and Natural Protein intake (R²=0.010)
Phenylpyruvic acid
Natural Protein intake
p = 0.483
Tartaric acid and Phenylalanine (R²=0.129)
Tartaric acid
phe (plasma)
p = 0.010
Tartaric acid and Natural Protein intake (R²=0.006)
Tartaric acid
Natural Protein intake
p = 0.575
Valine and Phenylalanine (R²=0.124)
Valine
phe (plasma)
p = 0.011
Valine and Natural Protein intake (R²=0.006)
Valine
Natural Protein intake
p = 0.598

Supplementary Figure S5. Multifactorial analysis of metabolites using standard coefficient of variation. Blue: not significant, orange: significant.

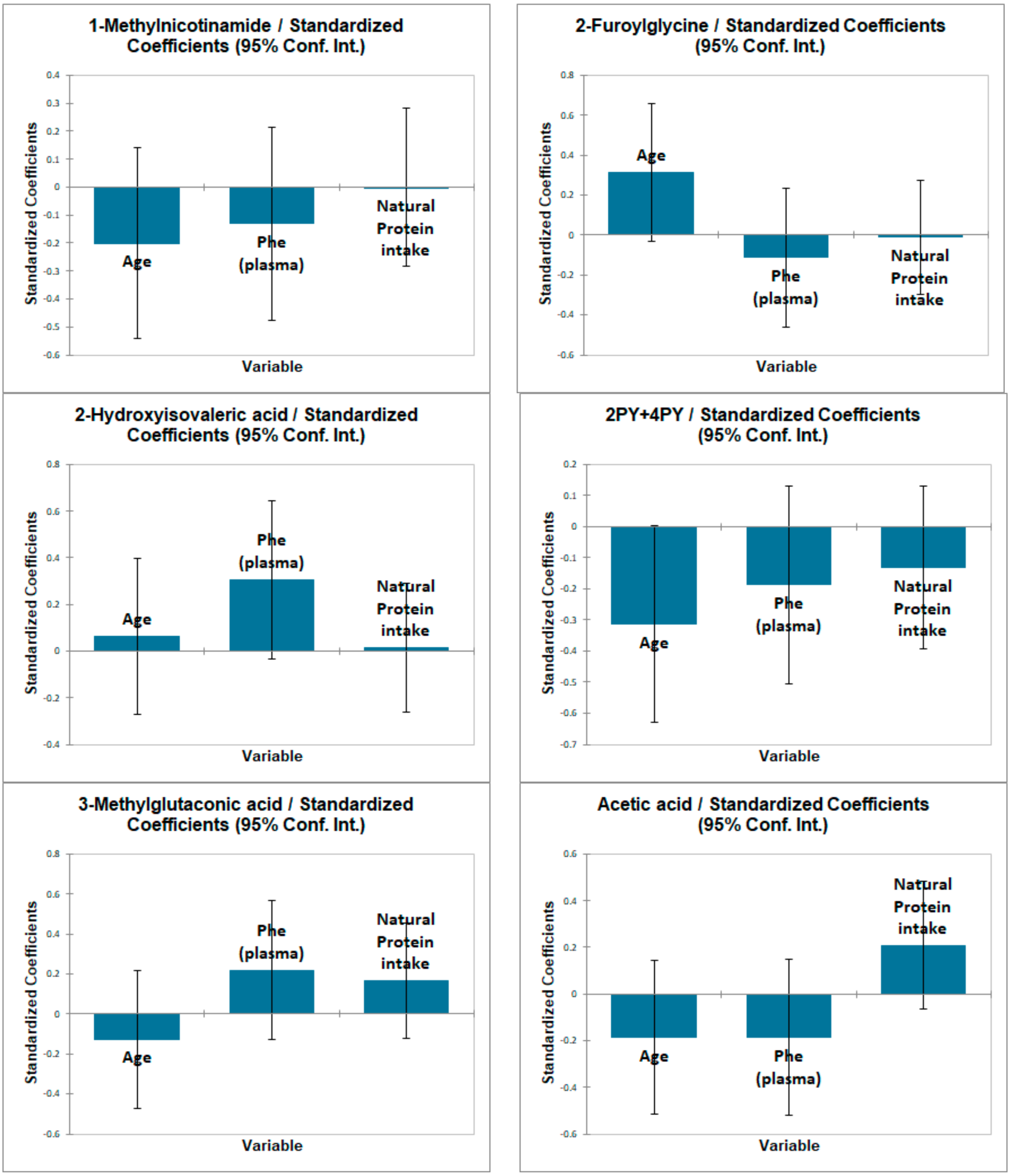

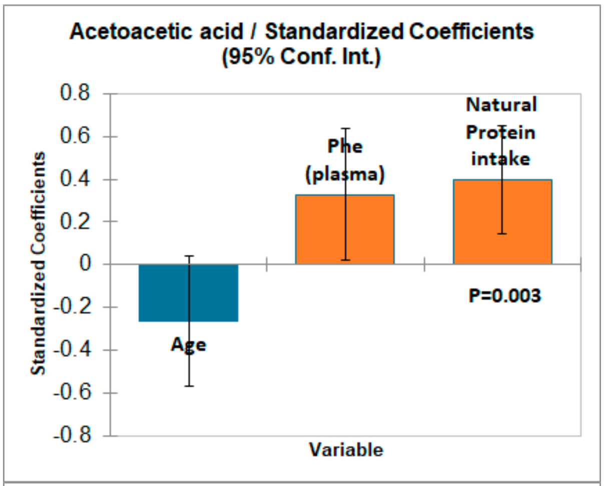
Acetoacetic acid / Standardized Coefficients (95% Conf. Int.)
Standardized Coefficients
0.8
0.6
0.4
0.2
0
-0.2
-0.4
-0.6
-0.8
Age
Phe (plasma)
Natural Protein intake
P=0.003
Variable

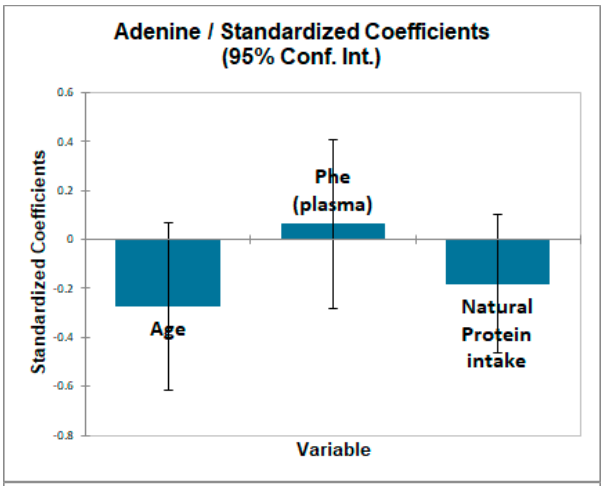
Adenine / Standardized Coefficients (95% Conf. Int.)
Standardized Coefficients
Age
Phe (plasma)
Natural Protein intake
Variable

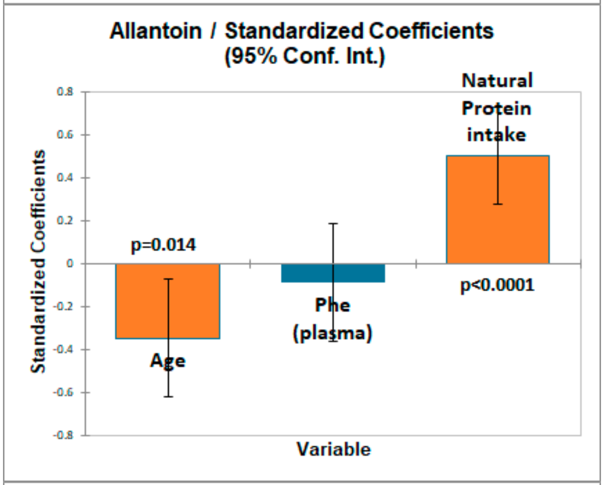
Allantoin / Standardized Coefficients (95% Conf. Int.)
Standardized Coefficients
p=0.014
Age
Phe (plasma)
Natural Protein intake
p<0.0001
Variable

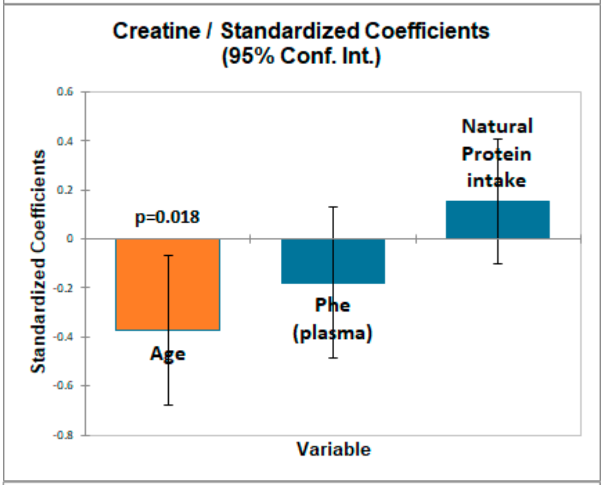
Creatine / Standardized Coefficients (95% Conf. Int.)
Standardized Coefficients
p=0.018
Age
Phe (plasma)
Natural Protein intake
Variable

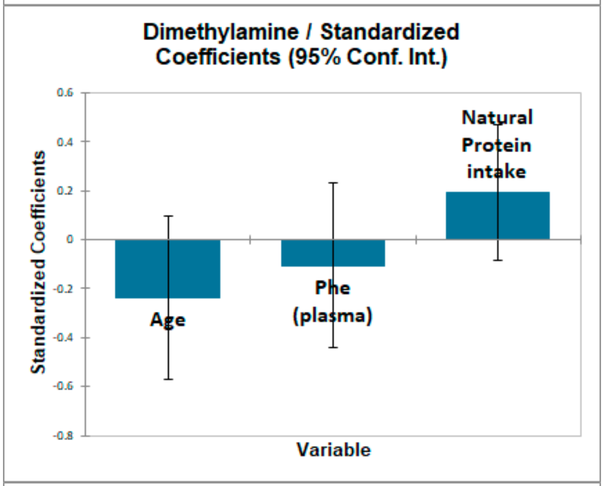
Dimethylamine / Standardized Coefficients (95% Conf. Int.)
Standardized Coefficients
Age
Phe (plasma)
Natural Protein intake
Variable

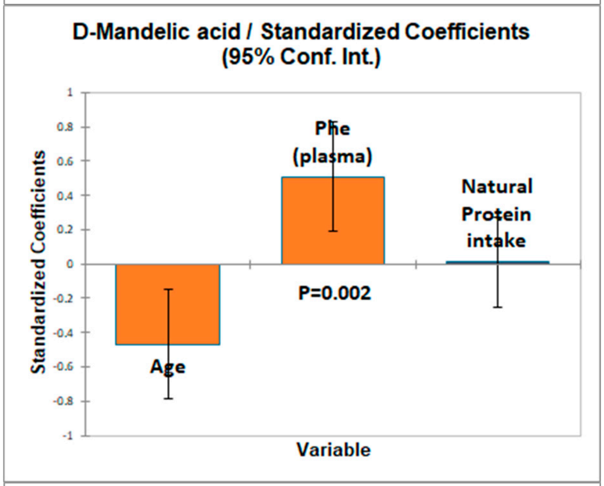
D-Mandelic acid / Standardized Coefficients (95% Conf. Int.)
Standardized Coefficients
Age
Phe (plasma)
P=0.002
Natural Protein intake
Variable

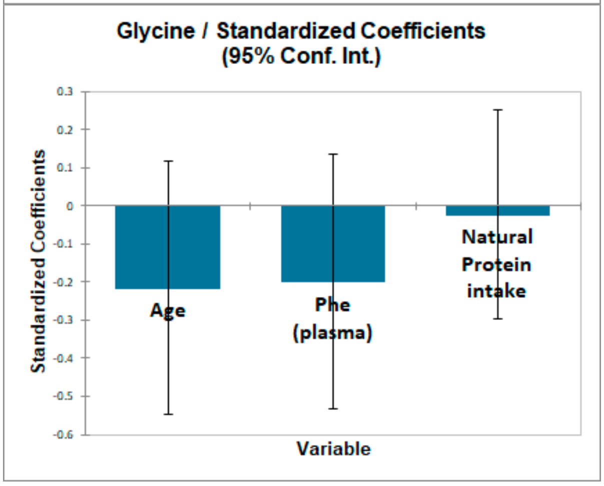
Glycine / Standardized Coefficients (95% Conf. Int.)
Standardized Coefficients
Age
Phe (plasma)
Natural Protein intake
Variable

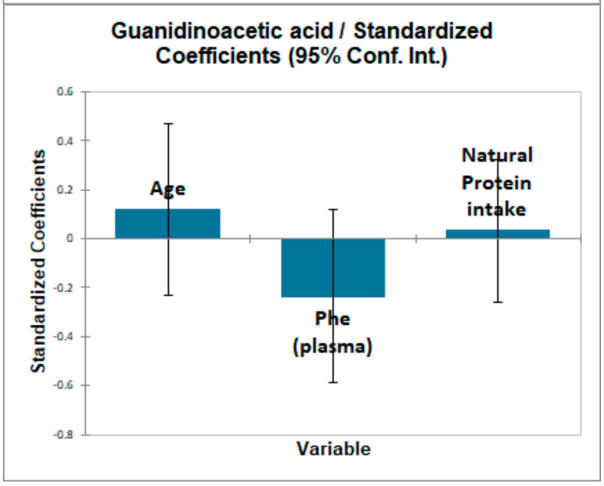
Guanidinoacetic acid / Standardized Coefficients (95% Conf. Int.)
Standardized Coefficients
Age
Phe (plasma)
Natural Protein intake
Variable

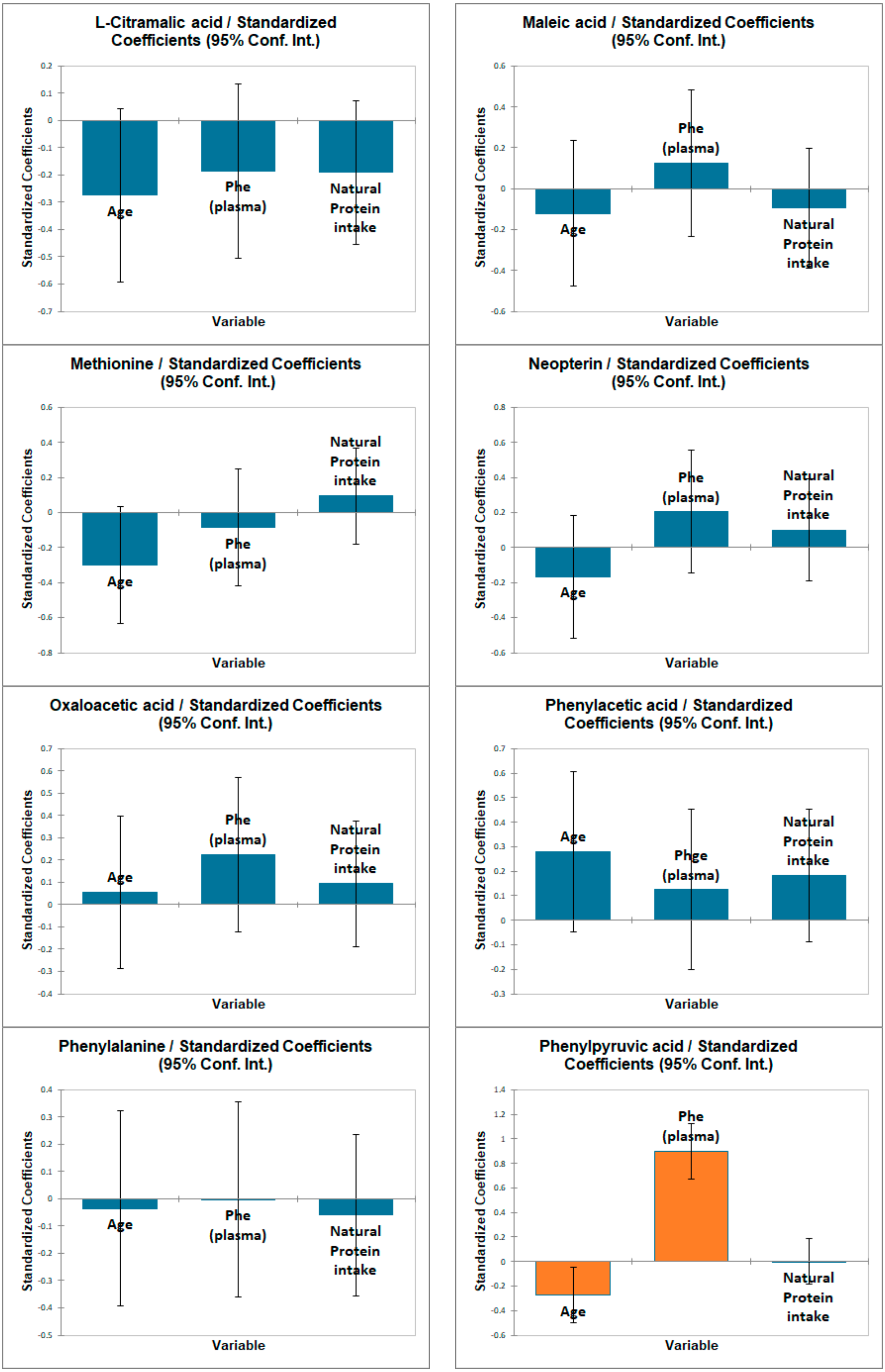
L-Citramalic acid / Standardized Coefficients (95% Conf. Int.)
Standardized Coefficients
Age
Phe (plasma)
Natural Protein intake
Variable
Maleic acid / Standardized Coefficients (95% Conf. Int.)
Standardized Coefficients
Age
Phe (plasma)
Natural Protein intake
Variable
Methionine / Standardized Coefficients (95% Conf. Int.)
Standardized Coefficients
Age
Phe (plasma)
Natural Protein intake
Variable
Neopterin / Standardized Coefficients (95% Conf. Int.)
Standardized Coefficients
Age
Phe (plasma)
Natural Protein intake
Variable
Oxaloacetic acid / Standardized Coefficients (95% Conf. Int.)
Standardized Coefficients
Age
Phe (plasma)
Natural Protein intake
Variable
Phenylacetic acid / Standardized Coefficients (95% Conf. Int.)
Standardized Coefficients
Age
Phge (plasma)
Natural Protein intake
Variable
Phenylalanine / Standardized Coefficients (95% Conf. Int.)
Standardized Coefficients
Age
Phe (plasma)
Natural Protein intake
Variable
Phenylpyruvic acid / Standardized Coefficients (95% Conf. Int.)
Standardized Coefficients
Age
Phe (plasma)
Natural Protein intake
Variable

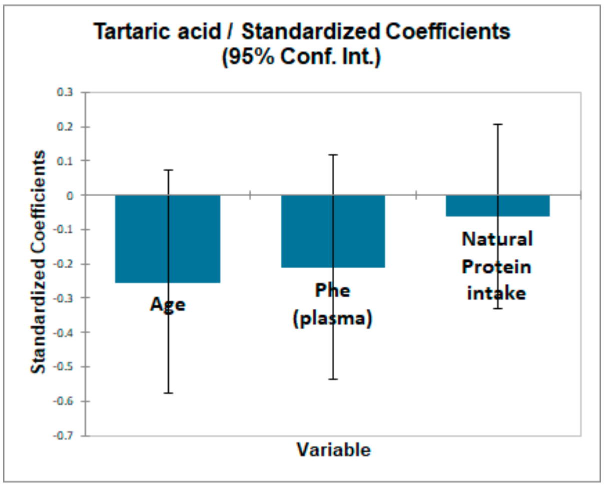

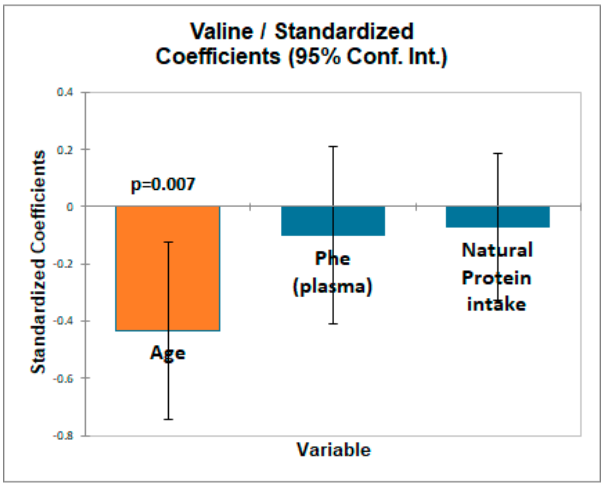

Supplemental text: Method for identification of unknown metabolites with UPLC-MS

Chemicals

Acetonitrile with 0.1% formic acid in LC-MS LiChrosolv quality was from Supelco. Water was prepared from a Milli-Q purification system from MerckMillipore. Ammonium acetate in LiChropure quality was from Supelco. Deuteriumoxide was from Deutero GmbH. N-Methyl-2-pyridone-5-carboxamide was from Sigma-Aldrich and N-Methyl-4-pyridone-3-carboxamide was from A2B Chem. Lithium formate was from Sigma-Aldrich.

Sample preparation

For UPLC-MS measurements, 50µL aliquot of each urine in the study pooled for QC sample. In general, 50µL of urine sample was diluted with 150µL Milli-Q water and transferred into a 96 well plate.

For the isolation of the unknown marker, the urine was evaporated for 2h at room temperature in order to improve the concentration. The injection volume was 10µL. After isolation, the fractions were evaporated to dryness and re-constituted in the HILIC mobile phase at starting conditions. For cleaning of the pre-isolated fractions, the injection volume was 10µL.

Instrumental

The UPLC System used for UPLC-MS-MS measurements and for pre-isolation and further cleaning of the fraction with HILIC chromatography was a Waters Acquity UPLC system (pump, sample manager, column manager, DA detector).

The column for LC-MSMS measurements was a Waters CORTECS T3 with a particle size of 2.7µm and a length of 100mm and an internal diameter of 3mm. The Mobile phase for LC-MSMS was A: water with 0.1% formic acid and B: acetonitrile with 0.1% formic acid. The flow rate was 0.25mL/min. The solvent gradient for LC-MSMS was: 0min, 100%A; 1.25min, 100%A; 6min, 90%A; 8min, 75%A; 10.5min, 25%A; 11min, 5%A; 11.5min, 5%A; 13.85min, 100%A.

The column for the pre-isolation step was a Waters BEH C18 with a particle size of 1.7µm, a length of 100mm and an internal diameter of 2.1mm. The mobile phase for the pre-isolation was A: water with 0.1% formic acid and B: acetonitrile with 0.1% formic acid. The flow rate was set to 0.25mL/min. The gradient for pre-isolation was: 0min, 100%A; 0.2min, 100%A; 12min, 60%A; 13min, 0%A; 14min, 0%A; 14.1min, 100%A; 16min, 100%A.

For cleaning of the pre-isolated fractions, an Xbridge HILIC column from Waters with a particle size of 3.5µm, a length of 100mm and an internal diameter of 4.6mm was used. The mobile phase for the cleaning step was: A: 90/10 ACN/100mM NH_4Ac and B: 50/40/10 ACN/H_2O/100mM NH_4Ac. The flow rate was set to 0.25mL/min. The solvent gradient for the cleaning step was: equilibration time 10min; 0min, 100%A; 5min, 100%A; 20min, 0%A; 25min, 0%A.

The mass spectrometer used for the detection of the unknown metabolites was a Bruker Impact II operated in positive ESI mode with a scan range from 50-1300 m/z. The calibrant used was 20mM lithium formiate.

Fractions were cut from the chromatography using a Valco multi position valve with 28 positions. The evaporation of the urine samples and peak cuts was done with a Thermo Speedvac SPD120 connected to a Vacuubrand MD 4C Vario select membrane pump. The evaporated samples were re-constituted with deuterium oxide and transferred to a 5mm NMR tube.

NMR spectra were acquired on a Bruker Avance NEO 600MHz spectrometer equipped with a 5mm helium cooled TCI probe head. 1D and 2D NMR experiments derived from CMC-se parameter sets were acquired. The structures of the unknown metabolites were calculated with the CMC-se structure elucidation software from Bruker Biospin. The results were confirmed by comparison of the spectra acquired for the unknown metabolites to the spectra of pure reference compounds purchased.
